# Allosteric pyruvate kinase-based “logic gate” synergistically senses energy and sugar levels in *Mycobacterium tuberculosis*

**DOI:** 10.1038/s41467-017-02086-y

**Published:** 2017-12-07

**Authors:** Wenhe Zhong, Liang Cui, Boon Chong Goh, Qixu Cai, Peiying Ho, Yok Hian Chionh, Meng Yuan, Abbas El Sahili, Linda A. Fothergill-Gilmore, Malcolm D. Walkinshaw, Julien Lescar, Peter C. Dedon

**Affiliations:** 1 Infectious Disease Interdisciplinary Research Group, Singapore-MIT Alliance for Research and Technology, 1 CREATE Way, Singapore, 138602 Singapore; 2 NTU Institute of Structural Biology, Nanyang Technological University, Singapore, 636921 Singapore; 3 Division of Life Science, State Key Laboratory of Molecular Neuroscience, Hong Kong University of Science and Technology, Clear Water Bay, Kowloon, Hong Kong China; 4 Institute of Quantitative Biology, Biochemistry and Biotechnology, University of Edinburgh, King’s Buildings, Edinburgh, EH9 3BF UK; 5 School of Biological Sciences, Nanyang Technological University, 60 Nanyang Drive, Singapore, 637551 Singapore; 6 Department of Biological Engineering, Massachusetts Institute of Technology, Cambridge, MA 02139 USA; 7 Present Address: Tychan Private Ltd, 80 Robinson Road, #17-02, Singapore, 068898 Singapore; 8 Present Address: Department of Integrative Structural and Computational Biology, The Scripps Research Institute, La Jolla, CA 92037 USA

## Abstract

Pyruvate kinase (PYK) is an essential glycolytic enzyme that controls glycolytic flux and is critical for ATP production in all organisms, with tight regulation by multiple metabolites. Yet the allosteric mechanisms governing PYK activity in bacterial pathogens are poorly understood. Here we report biochemical, structural and metabolomic evidence that *Mycobacterium tuberculosis* (Mtb) PYK uses AMP and glucose-6-phosphate (G6P) as synergistic allosteric activators that function as a molecular “OR logic gate” to tightly regulate energy and glucose metabolism. G6P was found to bind to a previously unknown site adjacent to the canonical site for AMP. Kinetic data and structural network analysis further show that AMP and G6P work synergistically as allosteric activators. Importantly, metabolome profiling in the Mtb surrogate, *Mycobacterium bovis* BCG, reveals significant changes in AMP and G6P levels during nutrient deprivation, which provides insights into how a PYK OR gate would function during the stress of Mtb infection.

## Abstract (web summary)

Pyruvate kinase (PYK) controls glycolytic flux. Here, the authors combine biochemical, structural and computational modelling studies to characterize the allosteric mechanisms regulating *Mycobacterium tuberculosis* PYK activity and show that AMP and glucose-6-phosphate are synergistic allosteric activators of the enzyme.

## Introduction

M*ycobacterium tuberculosis* (Mtb) is among the deadliest infectious diseases on a global scale, killing more than one-million people annually^1^, with emerging antimicrobial drug resistance posing serious challenges to existing diagnosis and treatment programs^1^. Although generally considered aerobic, Mtb has successfully adapted to the hypoxic and carbon-poor environment in human macrophages by evolving flexible carbon metabolism and co-catabolism to defend against the stresses posed by the human immune system^2–4^. In particular, increasing evidence suggests that the metabolic flexibility of central carbon metabolism (CCM: glycolysis, gluconeogenesis, pentose phosphate pathway and TCA pathway) is critical in Mtb physiology and pathogenicity^5–7^. This is illustrated by the rapid regulation of glycolytic activity in response to changes in ATP levels, which explains the enhanced efficacy of drug combinations that target the electron transport chain^8^. Here we describe a unique feature of the Mtb glycolytic enzyme, pyruvate kinase (PYK), which provides insights into its pivotal role in Mtb CCM^9^ and the metabolic flexibility of Mtb.

**Fig. 1:**
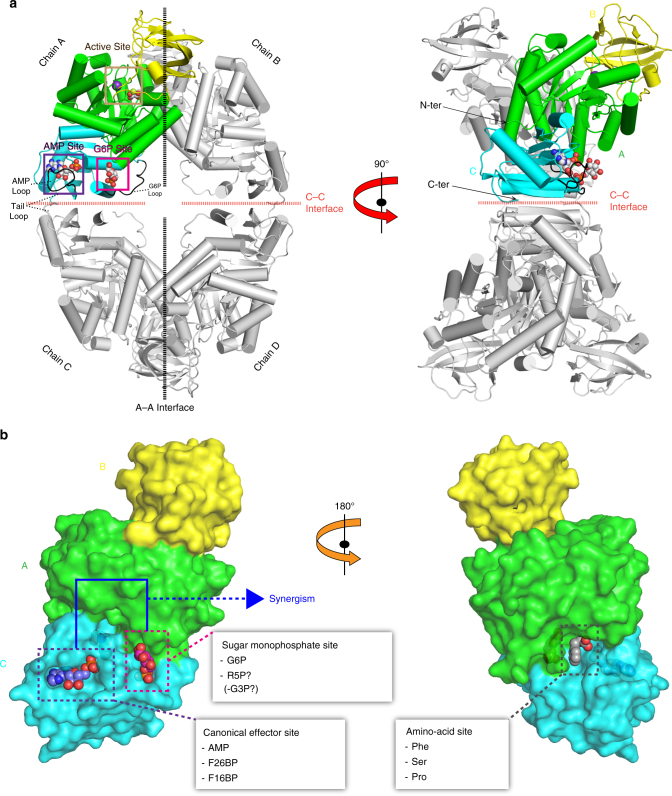
Structure of *Mtb*PYK and overview of three allosteric sites of PYK. **a** Crystal structure of the *Mtb*PYK-OX/AMP/G6P complex (PDB ID 5WSB) in the synergistically activated R-state. Two orthogonal views of *Mtb*PYK-OX/AMP/G6P show the tetramer architecture, domain boundaries, active site and synergistic effector sites. The A–A (large) and C–C (small) interfaces between subunits are shown as dashed lines. Each subunit comprises three domains, and one subunit (chain A) is coloured to show the domains: A-domain in green (residues 1–70, 168–336), B-domain in yellow (residues 71–167), C-domain in cyan (residues 337–472). The N terminus and C terminus of this subunit are indicated. Polypeptide chains are shown as cartoons, while metals and ligands are represented by spheres. Mg^2+^ and K^+^ located at the active site (brown box) are coloured in green and purple, respectively. The oxalate molecule (OX) at the active site is associated with the Mg^2+^. The canonical allosteric site (AMP-bound) is indicated by the purple box, while the newly discovered G6P-binding site (synergistically coordinating with the AMP-binding site) is shown by the magenta box. The AMP-binding loop (AMP loop) and G6P-binding loop (G6P loop) are coloured black. The C-terminal loop (tail loop) which undergoes a conformational change in the transition of inactive- and active states is indicated. **b** A surface representation of the PYK monomer (A-, B- and C-domains) highlighting three allosteric effector sites: canonical allosteric site that binds AMP, F26BP or F16BP; amino-acid site found in mammalian M1/M2PYK that binds amino acids as a nutrient sensor; sugar monophosphate site that binds G6P in *M. tuberculosis* synergistically coordinating with the canonical site, and probably binds R5P or G3P in some organisms. The A-, B- and C domains are shown in green, yellow and cyan, respectively. Effector-site ligands are shown as spheres

PYK (EC 2.7.1.40) forms a tetramer (Fig. 1a) that catalyzes transfer of phosphate from phosphoenolpyruvate (PEP) to ADP to form pyruvate and ATP. Given its central role in controlling glycolytic flux and ATP generation, PYK has been exploited as a drug target in bacterial pathogens^10^, parasites^11^ and cancer^12^, and was recently identified as a potential target of the antimalarial drug artemisinin^13^. The activity of most PYKs is tightly controlled by physiological modulators, with the notable exception of the constitutive activity of mammalian muscle isoenzyme M1. However, in spite of many decades of study, the complex allosteric mechanisms governing PYK activity in different organisms have eluded definition. For example, while fructose 2,6-bisphosphate (F26BP) allosterically activates trypanosome PYKs^14^, the upstream glycolytic intermediate, fructose 1,6-bisphosphate (F16BP), is the most widely recognised allosteric activator of PYKs in many bacteria^15, 16^, in yeast^17^ and in mammals^18^. In general, PYKs from higher organisms have a single essential modulator, with the exception of F16BP and amino-acid regulation of human M2PYK in cancer-cell proliferation^19–21^. This stands in contrast to the many bacterial PYKs that use ‘non-canonical’ effectors such as AMP and the sugar monophosphates glucose 6-phosphate (G6P) and ribose 5-phosphate (R5P) for allosteric regulation (Supplementary Table 1), including PYKs from important human pathogens such as Mtb^9^, *Streptococcus mutans* ^22^, *Staphylococcus aureus* ^23^ and *Salmonella typhimurium* ^16^ (a sequence alignment of selected bacterial PYKs is shown in Supplementary Fig. 1 and pairwise identities in Supplementary Table 2).

One of the reasons that the allosteric mechanisms regulating bacterial PYKs have remained elusive for decades is the lack of structural information about the binding of physiological effectors to identify allosteric sites. Here we report biochemical, structural and computational modelling studies of Mtb PYK that reveal a ‘rock-shape-lock’ allosteric mechanism regulated by the synergistically acting activators AMP and G6P. Stress-induced metabolomic changes in the Mtb surrogate, *Mycobacterium bovis* BCG, point to AMP and G6P as molecular input signals that position *Mtb*PYK as a unique molecular OR ‘logic gate’ to sense changes in energy and sugar levels during Mtb infection.

## Results

### Effectors AMP and G6P activate *Mtb*PYK synergistically

We first performed detailed kinetic studies of purified *Mtb*PYK to evaluate AMP and G6P as allosteric activators and to test whether they share a single binding site (Table 1). *Mtb*PYK purified from an *Escherichia coli* expression system was more active than that reported by Noy et al.^9^ under similar assay conditions, with a *k*_cat_ value of 183 ± 1 s^−1^ versus 63 s^−1^, respectively (Supplementary Table 1), and it showed hyperbolic kinetics with respect to its substrate ADP, with a *K*_m_ value of 0.47 ± 0.02 mM. For the substrate PEP, the enzyme displayed sigmoidal kinetics in the absence of effector, with a *S*_0.5_ value of 0.41 ± 0.01 mM and a Hill coefficient (*h*) of 1.82 ± 0.05, indicating positive cooperativity. The affinity to substrate PEP was estimated by its *S*_0.5_ value in the presence of saturating ADP. Therefore, both AMP and G6P enhanced the affinity of PEP by ~2-fold and decreased the cooperativity with respect to PEP (*h = *~1.2). Inhibition by the product ATP (Supplementary Fig. 2) reduced PEP affinity by 5-fold (*S*_0.5 = _2.20 ± 0.08 mM) and increased the cooperativity to PEP (*h* = ~2.6). ATP inhibition was reversed by adding the allosteric activators AMP and G6P.

**Table 1:** Kinetic properties of *Mtb*PYK

Ligand	Kinetic parameter				Modulators		
		None	+AMP^a^	+G6P^a^	+AMP/G6P^a^	+ATP^a^	+ATP/AMP/G6P^a^
PEP	*S*_0.5_ (mM)	0.41 ± 0.01	0.25 ± 0.01	0.18 ± 0.01	0.18 ± 0.01	2.20 ± 0.08	0.37 ± 0.01
	*h*	1.82 ± 0.05	1.21 ± 0.04	1.24 ± 0.07	1.23 ± 0.03	2.63 ± 0.21	1.15 ± 0.03
	*k*_cat_ (s^−1^)	183 ± 1.2	194.8 ± 2.1	172.7 ± 2.8	188.8 ± 1.4	101 ± 2.7	136 ± 1.3
	*k*_cat_/*S*_0.5_	446	780	960	1049	46	367
ADP	*K*_m_ (mM)	0.47 ± 0.02	N.A.				
		None	+ AMP		+ G6P		
			12.5 µM	25 µM	25 µM	50 µM	
AMP	*K*a_0.5_ (µM)	63.5 ± 2.9	N.A.	N.A.	35.7 ± 0.9	20.3 ± 1.4	
G6P	*K*a_0.5_ (µM)	147 ± 5.8	55.4 ± 3.1	42.2 ± 3.4	N.A.	N.A.	

*k*_cat_/*S*_0.5_ values in s^−1^ mM^−1^

*h* Hill coefficient, N.A. not applicable

^a^The concentrations of modulators in the kinetic study of PEP are: AMP—1 mM, G6P—1 mM, ATP—2 mM

The concentrations of AMP and G6P were then varied to investigate their interplay in enzyme activation (Table 1). The *K*_a0.5_ value of AMP fell up to 3.2-fold (~64 to ~20 µM) as the concentration of G6P was increased. In a similar manner, the apparent affinity of G6P increased up to 3.5-fold (~147 to ~42 µM) with increasing AMP. This positive cooperativity between AMP and G6P suggests two distinct binding sites instead of competitive binding to the same effector site. In addition, the enzyme catalytic efficiency, which is estimated by *k*_cat_/*S*_0.5_ value (Table 1), is also enhanced by the synergy of AMP and G6P. In contrast to previous computational studies in which effectors were docked at a single canonical allosteric site of bacterial PYKs^24^, our kinetic results demonstrate that AMP and G6P act in synergy to allosterically activate *Mtb*PYK and suggest two cooperative allosteric sites. The latter was borne out in structural studies.

### A rigid-body rocking motion in allosteric transition

More than 40 years ago, Waygood et al.^25^ demonstrated for the first time that AMP and G6P were the activators of PYK using the *E. coli* enzyme as the model. However, AMP- or G6P-bound PYK structures have been hitherto unavailable to elucidate the corresponding effector sites and allosteric mechanisms. Here, we have successfully determined a series of six high-resolution crystal structures of PYK from *M. tuberculosis* in both inactive T-state and active R-state, and with or without AMP and G6P (Table 2).

**Table 2:** Data collection and refinement statistics

	T-state *Mtb*PYK	*Mtb*PYK-OX	*Mtb*PYK-OX/ATP/AMP	*Mtb*PYK-OX/G6P	*Mtb*PYK-OX/AMP/G6P	*Mtb*PYK- OX/AMP/G6P (soak)
PDB ID	5WRP	5WS8	5WS9	5WSA	5WSB	5WSC
*Data collection*						
Space group	P2_1_2_1_2_1_	P3_1_	P3_1_	P3_1_	P3_1_	P3_1_
Cell dimensions						
*a*, *b*, *c* (Å)	90.76,129.48,243.98	127.01,127.01,144.65	127.19,127.19,144.92	125.42,125.42,144.19	125.63,125.63,144.51	124.37,124.37,144.19
*α*, *β*, *γ* (°)	90.00,90.00,90.00	90.00,90.00,120.00	90.00,90.00,120.00	90.00,90.00,120.00	90.00,90.00,120.00	90.00,90.00,120.00
Solvent content (%)	65	63	63	62	62	61
Resolution (Å)	88.79–2.85	63.51–2.62	60.54–1.90	62.71–2.85	62.82–2.25	53.85–2.40
No. of reflections	271,505 (15,837)	432,224 (25,144)	1,001,199 (43,594)	333,176 (25,316)	616,913 (15,502)	541,318 (24,538)
No. of unique reflections	67,441 (4498)	78,513 (4558)	206,700 (10,249)	59,240 (4587)	120,301 (5497)	97,539 (4826)
Wilson B-factor (Å^2^)	55.070	50.351	23.261	37.737	31.826	24.399
*R*_merge_ (%)	11.4 (92.7)	9.4 (75.6)	7.1 (44.6)	10.3 (45.5)	10.0 (89.9)	17.4 (86.8)
*I*/*σI*	7.9 (1.1)	10.6 (2.2)	12.0 (3.0)	13.7 (3.3)	9.9 (1.4)	8.3 (1.6)
Completeness (%)	99.4 (99.9)	100.0 (100.0)	100.0 (100.0)	100.0 (100.0)	99.3 (92.1)	100.0 (99.9)
Multiplicity	4.0 (3.5)	5.5 (5.5)	4.8 (4.3)	5.6 (5.5)	5.1 (2.8)	5.5 (5.1)
*Refinement*						
Monomers in ASU	4	4	4	4	4	4
No. of reflections	63,878	74,461	195,933	56,202	114,312	92,706
*R*_work_/*R*_free_	0.2056/0.2330	0.1734/0.2178	0.1968/0.2229	0.1509/0.1953	0.1870/0.2278	0.1793/0.2270
No. of non-H atoms						
Protein	13549	14044	14211	14172	14172	14172
Water	234	494	1440	172	410	335
Ligands	20	28	269	112	195	195
Average B-factor (Å^2^)						
Protein	76.0	70.2	36.3	43.6	37.4	28.2
Water	55.8	57.7	43.4	24.3	27.2	22.1
Ligands	76.8	65.9	47.4	32.8	31.1	23.4
AMP/G6P	N.A.	N.A.	30.0	33.1	30.9	23.6
RMS deviations						
Bond lengths (Å)	0.0123	0.0117	0.0121	0.0143	0.0119	0.0140
Bond angles (°)	1.1760	1.1809	1.2636	1.3289	1.3243	1.5284
Ramachandran plots						
Favoured (%)	97.1	96.7	98.3	97.2	97.3	97.8
Allowed (%)	99.8	99.8	99.6	99.8	99.6	99.6
Number of outliers	4	4	8	4	8	8

Values in parentheses are for the highest resolution shell

N.A. not applicable

*Mtb*PYK adopts a tetrameric architecture formed by identical subunits with three domains (A, B and C domains) (Fig. 1a), similar to typical PYK structures. The catalytic site is located in the cleft between the A and B domains (lid domain), while the C domain harbours a canonical AMP allosteric site. The allosteric site binding for G6P (sugar monophosphate site) is adjacent to the AMP site and lies between the A and C domains (Fig. 1b). It is noteworthy that the amino-acid binding site of human M2PYK is also formed by the A and C domains but is located on the opposite pole of the molecule relative to the canonical allosteric site (Fig. 1b).

**Fig. 2:**
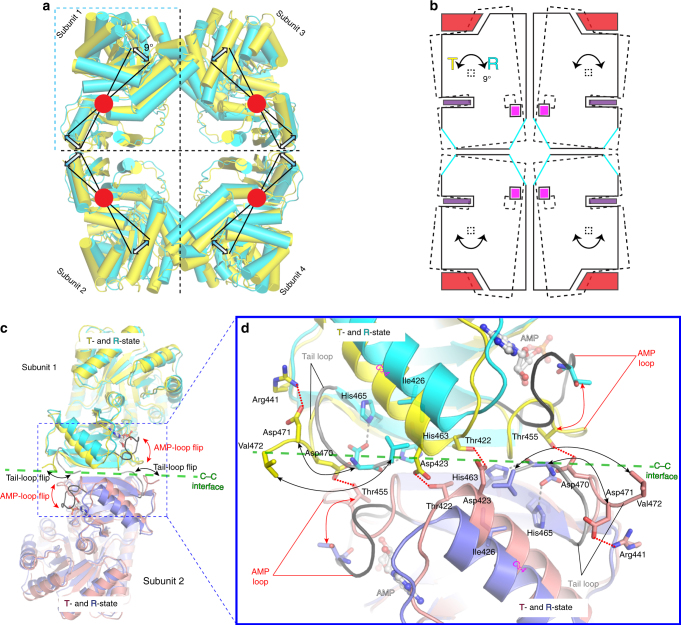
T- to R-state transition requires the disruption of C–C interface interactions and is enhanced by effector binding. **a** Rigid-body rotation showing the transition between T- and R-states of *Mtb*PYK. The C-α atoms of the AC cores (A- and C domains) of the inactive T-state tetramer (yellow) were superposed onto the active R-state tetramer (cyan). The superposed polypeptide chains are shown as cartoons, and B-domains have been removed for clarity. The T- (yellow) and R-state (cyan) transition is represented by a 9° rigid-body (AC core) rotation around the central pivot (indicated by the red circle). **b** Schematic representation of the rigid-body rotation of the AC cores between the T- (dashed lines) and R-states (solid lines). Ligands AMP, G6P and oxalate are shown as purple rectangle, magenta square and red trapezoid, respectively. The direction of movement is shown using arrows. The structural reshaping of the allosteric sites induced by AMP/G6P synergism is indicated in cyan. **c** Side view of the superposed tetramers of T-state *Mtb*PYK and R-state *Mtb*PYK-OX/AMP/G6P. The AC cores of two tetramers were superposed (C-α atoms fit). The polypeptide chain is shown as a cartoon while effectors are shown as sticks. The B domains have been removed for clarity. Only two subunits are shown: subunit 1 and subunit 2. The C-C interface formed between subunit 1 (T-state in yellow and R-state in cyan) and subunit 2 (T-state in salmon and R-state in blue) is indicated using a green dashed line. Interface loops are indicated and the flips of loops between T- and R-states are shown by arrows. In the R-state, AMP loops and tail loops are indicated in black and grey, respectively. **d** Enlargement of the C–C interface indicating the conformational changes and the rearrangement of interface interactions. The movements of loops and residues are indicated by arrows. Interactions in the T-state structure are shown as red dotted lines, while interactions in the R-state structure are shown as grey dashed lines

The allosteric mechanism was analysed at the level of quaternary protein structure by superposition of the inactive T-state tetramer structure (PDB: 5WRP) onto the fully ligated R-state tetramer structure (*Mtb*PYK-OX/AMP/G6P; PDB: 5WSB) excluding the mobile B domains. This gave an RMS fit of 3.2 Å for all C-α atoms. The superposition analysis suggests that each subunit of the *Mtb*PYK tetramer simultaneously undergoes a 9° AC-core (A and C domains) rigid-body rotation concomitant with the T- to R-state transition (Fig. 2a, b; Supplementary Table 3), which is consistent with the ‘rocking motion’ mechanism observed in trypanosomatid PYKs^14, 26, 27^ and human M2PYK^19^. We have also compared the rigid-body rotation angles from T-state to different ligated R states of the *Mtb*PYK tetramer (Supplementary Table 3), and observed similar motion angles (<1° variation). Our results provide the detailed structural evidence that bacterial PYKs possess a concerted ‘rocking motion’ allosteric mechanism (Supplementary Movie 1), despite having a distinct repertoire of allosteric effectors.

### The interplay between AMP and C-terminal tail loops

We next explored the determinants of this concerted rigid-body rotations of all four chains during the allosteric interconversion of T- and R-states in the *Mtb*PYK tetramer (Fig. 2a, b). Unlike trypanosomatid PYKs that use additional salt bridges across the C–C interface to stabilise the R-state tetramer structure^14, 26, 27^, the motions of the AC-cores in *Mtb*PYK require the disruption of C–C interface interactions between the AMP loop and the C-terminal tail loop (Thr455…Asp470), as well as the hydrogen bonds (H-bonds) formed between the two adjacent Cα4 structures (Thr422…Asp423) (Fig. 2c, d). The electron density of the AMP loop is poorly identified in the R-state *Mtb*PYK-OX structure (PDB: 5WS8) indicating high flexibility in the absence of effector binding.

The relocation of the AMP loop leaves a free space at the C–C interface and thus allows the reorganisation of the C-terminal tail loop (Fig. 2c). This tail loop flips ~180° to approach the C–C interface and forms H-bonds with the Cβ4 strand from the same protein chain (Asp471…His463/His465) while the hydrophobic residue Val472 (the C-terminal portion of the tail loop) forms a hydrophobic interaction with the side-chain of Ile426 in helix Cα4 (Fig. 2d). This ‘in-and-out’ interplay between the AMP loop and the C-terminal tail loop, in response to the transition between T- and R-states, favours the formation of the R conformation by abolishing interface H-bonds and generating new interactions within each protomer (Supplementary Movie 2). The loss of interface interactions is also in agreement with the reduction in the C–C interface area in the R-state (Supplementary Table 4).

The thermal stability of *Mtb*PYK in all possible ligated states was determined in the presence of a wide variety of ligands by performing fluorescence-based thermal shift assays (Supplementary Fig. 3). When one or more allosteric ligands were mixed with the enzyme in the presence of substrate PEP or oxalate, the thermal vibration decreased (Δ*T*_m_ = 1–10 °C), suggesting that the stability of the R conformation is enhanced by a network of interactions between allosteric ligands and each protein subunit, which overcomes the reduction in C–C interface interactions. Kinetic evidence of enzyme activation by AMP and G6P correlates well with the enhanced rigidity of the R-state PYK in the presence of AMP and G6P. The physical AMP and G6P binding sites were established next, with the discovery of the G6P-binding site.

### AMP binds at the canonical allosteric site

**Fig. 3:**
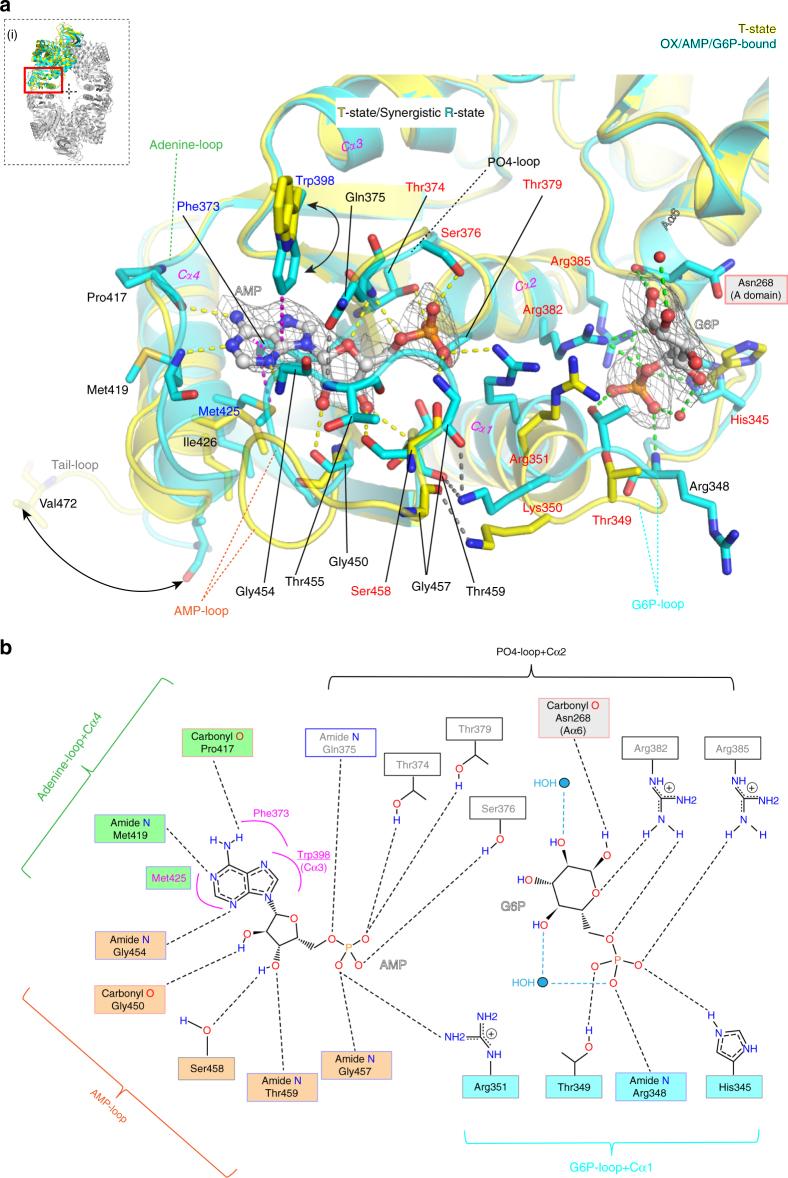
AMP and G6P bind at two distinct allosteric sites. **a** Close-up view of the superposed allosteric sites of T-state *Mtb*PYK (yellow) and R-state *Mtb*PYK-OX/AMP/G6P (cyan). The polypeptide chain is shown as a cartoon while interacting residues are shown as sticks. Allosteric effectors AMP and G6P are shown with an unbiased *Fo–Fc* electron-density map contoured at 3.0 *σ* (grey). Water molecules are shown as red spheres. Interactions between ligands and the R-state structure are indicated by dashed lines. The T-shaped stacking (or CH–π hydrogen bonding) interactions formed between the adenine ring of AMP and *Mtb*PYK residues (Phe373, Trp398, Met425) are shown by pink dashed lines. Secondary structures that are involved in the interactions with effectors are indicated. The conformational changes of the C-terminal tail loop and the side-chain of residue Trp398 are indicated by arrows. The location of the allosteric site within a subunit is shown as a red box in the inset (i). **b**, Schematic drawing showing the synergistic interactions at the *Mtb*PYK allosteric sites. Residues forming T-shaped stacking (or CH–π hydrogen bonding) interactions with the adenine ring of AMP are indicated in pink, while water molecules are shown as blue circles

While AMP binds tightly at the canonical allosteric site of PYK (Fig. 3) that recruits bisphosphate effectors such as F16BP^17, 18^ and F26BP^14^ in many organisms, the *Mtb*PYK AMP binding site has several unique features. In *Mtb*PYK, the AMP phospho group is H-bonded to residues Thr374, Gln375, Ser376, Thr379 and AMP-loop residue Gly457, and forms a salt bridge with the side-chain of Arg351 in helix Cα1. By comparison with the AMP-free T-state structure (PDB: 5WRP), the effector loop (residues 451–458) moves further from the dimer–dimer (C–C) interface to wrap around AMP and form H-bonds with it. The adenine-binding site for AMP is relatively hydrophobic and is composed of residues Phe373, Trp398 and Met425. These residues form stacking interactions with the adenine ring of AMP (Supplementary Fig. 4). Interestingly, the binding of Trp398 to AMP requires reorientation of the side chain of this residue to close up the AMP pocket. The fact that Trp398 is not highly conserved in other AMP-activated PYKs (Supplementary Fig. 1) suggests a unique regulatory function in *Mtb*PYK. Hydrophobic residues Phe373 and Met425 are relatively conserved among PYKs that use AMP as activator. Residue Met425 in helix Cα4 is normally replaced by arginine in F16BP- or F26BP-activated PYKs^14, 17, 18^ (Supplementary Fig. 5a), whereby a positively charged side chain is essential to form a salt bridge with the negatively-charged 1′- or 2′-phospho group to hold the effector in place. Moreover, Gln375 in *Mtb*PYK is substituted by a positively charged lysine residue in human M2PYK which interacts with the 1′-phospho group of F16BP via a salt bridge^18^ (Supplementary Fig. 5a). Therefore, the hydrophobicity of the adenine-binding site in the AMP pocket is strictly required for recognition. By contrast, in F16BP- or F26BP-activated PYKs, the effector site needs to provide more positive charges (Arg, Lys substitutions) to recognise and lock both negatively charged phospho groups in place.

### G6P binds at an allosteric site adjacent to canonical site

The kinetics-based prediction of a unique G6P-binding site was borne out in the structural analyses. The *Mtb*PYK structures in complex with G6P reveal an allosteric site that is distinct from the canonical AMP-binding allosteric site (Fig. 3). The G6P site is located at the boundary between the A and C domains (Fig. 1), and is composed of helices Aα6, Cα1, Cα2 and the G6P-loop (Fig. 3). Two positively-charged residues Arg382 and Arg385 in Cα2 lock the G6P phospho group in place via salt bridges together with three H-bonds provided by three residues in the G6P-loop (His345, Arg348, Thr349). Interestingly, the side-chain of His345 in the G6P loop adopts multiple positions within a tetramer in the absence of G6P binding, but is locked in one position by H-bonding to G6P. The sugar group of G6P makes a H-bond with the main-chain of Asn268 in Aα6. Water molecules further stabilise G6P. In contrast, the corresponding site in non-G6P-regulated PYKs (i.e., trypanosome PYK, human M2PYK) has more negatively-charged residues that could prevent G6P binding (Supplementary Fig. 5b).

Structurally, Cα1 bridges the AMP and G6P sites, where Arg351 in Cα1 forms an ionic pair with AMP and Thr349 (next to Cα1) interacts with the G6P phospho group by H-bonding. The presence of this prominent structure network of AMP…Cα1…G6P correlates well with the kinetic synergy between AMP and G6P. These results suggest the existence of two physical allosteric pathways linking the AMP and G6P binding sites with the active site ~40 Å away, which was established next.

### Dual allosteric pathways with synergistic cooperation

**Fig. 4:**
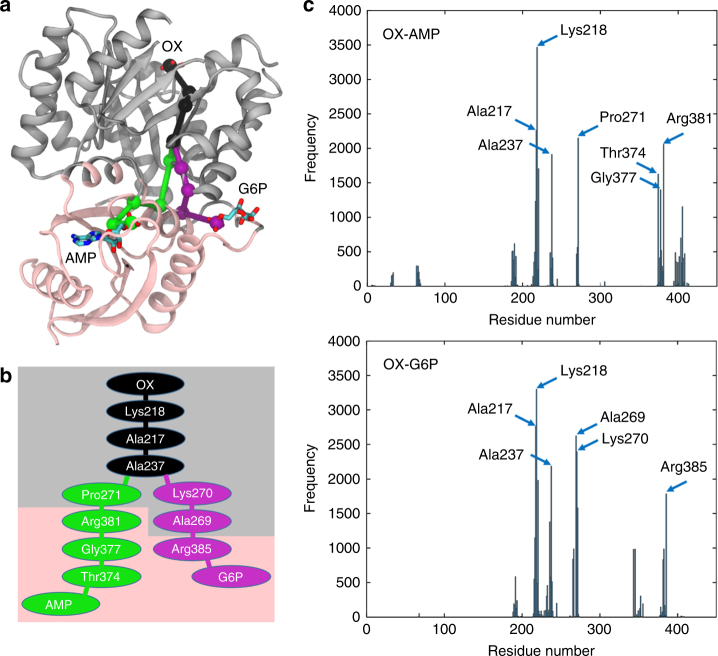
Allosteric pathways between the catalytic site and the two allosteric sites. **a** The allosteric pathways of OX-AMP (green) and OX-G6P (purple) share three common residues, namely Ala217, Ala237 and Lys218 (black spheres). The shared portion of the pathway is coloured black. The protein is drawn in ribbon and coloured according to community analysis. Note that the lid B domain is not shown for clarity. **b** A schematic shows the two allosteric pathways and the key participating residues. The residues could be grouped in two different communities, coloured in grey and pink. **c** Histograms of the key residues involved in the pathways, showing the frequency at which a particular residue was identified in one of the 4000 calculated pathways. The allosteric pathways were extracted from the MD simulations using WISP dynamic network analysis

To explore the physical basis for this dual allostery, we performed a molecular dynamics (MD) simulation on the tetramer of *Mtb*PYK with bound OX/AMP/G6P in order to identify potential allosteric pathways between the catalytic site (OX site) and the two allosteric sites (AMP and G6P sites). The allosteric pathways between oxalate and AMP/G6P were then extracted from the MD simulation using the Weighted Implementation of Suboptimal Pathways (WISP)^28^. Dominant allosteric pathways connecting oxalate and AMP (OX-AMP), as well as connecting oxalate and G6P (OX-G6P), are shown in Fig. 4a, b. An allosteric pathway was considered dominant when it consisted of a set of residues with high frequency of occurrence in the histogram shown in Fig. 4c. The two allosteric pathways propagate through three identical residues en route to the catalytic site, namely Ala237, Ala217 and Lys218, which suggests that synergistic cooperation exists, perhaps at the level of individual *Mtb*PYK monomers. Further community analysis revealed that AMP and G6P-binding sites belong to the same group of highly correlated residues (Supplementary Fig. 9), suggesting binding of either activator could allosterically regulate the catalytic binding site. This was explored next.

### A synergistic allostery beyond rock-and-lock

**Fig. 5:**
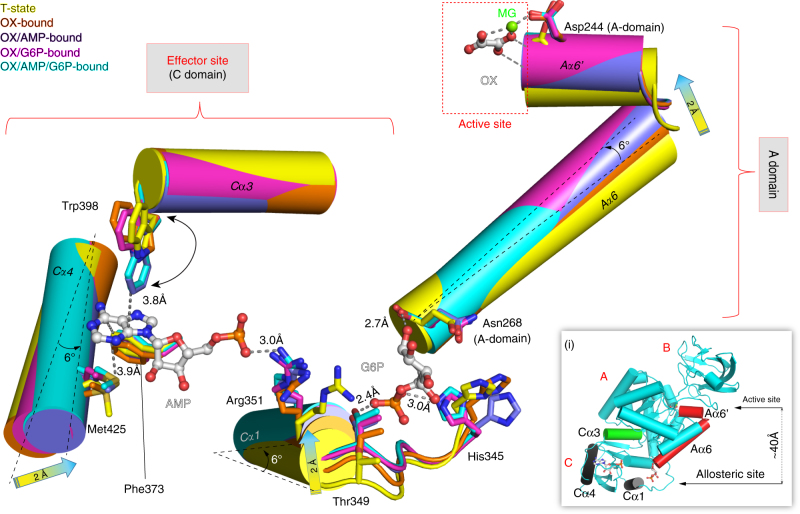
Local conformational rearrangements within the C domain induced by the effectors binding in a synergistic manner. Effector-site superposition of five *Mtb*PYK structures in different ligand-bound states showing the structural rearrangements of the synergistic mechanism. The polypeptide chains are shown as cartoons, while interacting residues are shown as sticks. The carbon atoms of the effectors AMP and G6P are represented by grey sticks. The movements of α helixes (Cα1, Cα4 and Aα6′–Aα6) and the flip of the side chain of Trp398 are indicated by arrows. Interactions between *Mtb*PYK and the effectors are shown as dashed lines, together with the corresponding distances. The relative locations of the five implicated α helixes within a subunit are shown in the inset (i) where the active site, allosteric effector-binding site and domains are indicated. The α helixes Cα3 and Aα6′–Aα6 are shown as green and red, respectively. The distance between the active site and effector is about 40 Å

Studies of PYKs from trypanosomatids^14^ and humans^19^ have established an allosteric ‘rock-and-lock’ regulation mechanism involving rigid-body rotations of each subunit in the tetramer. We have demonstrated this in *Mtb*PYK (Fig. 2). However, the conformational flexibility within each subunit of the tetramer has not yet been reported, except for the flexible effector loop and the mobile B domain^27^ (Supplementary Fig. 6; Supplementary Table 5). To study this type of structural plasticity and to probe the mobile secondary structural elements (α helix, β strand, loops), we superposed monomers (instead of tetramers) of all available R-state *Mtb*PYK structures onto the T-state monomer structure. The mobile B domain and AMP loop were excluded from the superposition. The distances between Cα-carbons following superposition ranged from 0.7 to 1.1 Å (Supplementary Table 3), which indicates only a subtle change in structure. We then further analysed the superposition data by plotting a heat-map using the Cα distance of each pair of superposed atoms (Supplementary Fig. 7). The heat-map clearly reveals correlated local structural rearrangements (shown in red) in response to the concerted rigid-body motion and the AMP/G6P synergistic binding. This indicates the ‘plastic’ nature of a *Mtb*PYK monomer, as illustrated in Fig. 5 and Supplementary Movie 3.

### AMP/G6P kinetic synergy results from a synergistic allostery

A more granular analysis of the allosteric site reshaping upon AMP and G6P binding revealed a unique mechanism underlying the AMP/G6P kinetic synergy. Cα1 and Cα4 are essential α helices at the two allosteric sites of *Mtb*PYK that regulate the binding of G6P and AMP, respectively (Fig. 3). Both exhibit obvious movements during the T- and R-state transition (Fig. 5; Supplementary Fig. 7). This structural plasticity of allosteric sites in response to effector binding has not been reported in any other PYK. We then further analysed the movements of superposed structures and found that Cα1 and Cα4 both rotated and shifted by ~6° from the T-state (yellow) to the fully ligated R-state (cyan) (Fig. 5). In contrast, other non-fully ligated *Mtb*PYK R-state structures exhibit less obvious but similar Cα1/Cα4 motion during the T- and R-state interconversion. Briefly, as shown in Fig. 5, only a subtle movement of Cα1 is observed between the T- (yellow) and oxalate-bound R-state (*Mtb*PYK-OX; orange), with Cα4 remaining static. AMP binding (purple) triggers nearly maximum Cα4 movement (~6°) but modest Cα1 movement. By contrast, G6P binding (magenta) causes Cα1 to shift nearly maximally (~6°), but with only a subtle movement of Cα4. To further confirm the structural plasticity of *Mtb*PYK allosteric sites, we soaked AMP and G6P into *Mtb*PYK-OX crystals resulting in nearly identical allosteric sites (Cα RMS fit: 0.17 Å) compared with that of the fully ligated R-state structure determined from the crystal using a co-crystallization method. Both fully ligated R-state structures, obtained from distinct crystallization methods, display the same degree of Cα1/Cα4 movement (~6°) during the T- and R-state transition, further demonstrating the dramatic plasticity of the allosteric-site structures regulated by effectors.

**Fig. 6:**
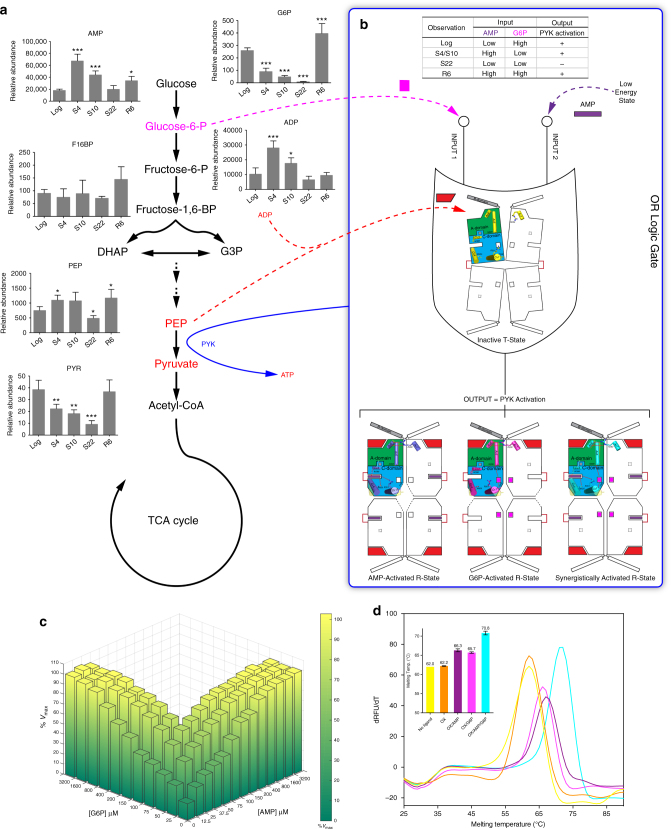
Allosteric PYK-based molecular OR logic gate synergistically regulates energy and carbon metabolism in mycobacteria. Histograms of metabolic changes at S4, S10, S22 and R6 against Log in nutrient-starvation model; abundance data were normalised to protein concentration and represent mean ± SD, *n* = 4; significance is indicated as *p < 0.05, **p < 0.01, ***p < 0.001 by one-way ANOVA with Dunnett post test versus Log. **a** Schematic illustration of glycolysis pathway and metabolic changes in *M. bovis* BCG during nutrient starvation. The allosteric activator of *Mtb*PYK, glucose-6-P (G6P), is shown in magenta, while PYK substrates (ADP and PEP) and products (ATP and pyruvate) are coloured in red. **b** A schematic representation of the molecular OR gate. The metabolite G6P (magenta square) and the low-energy-state signal AMP (purple rectangle) are two molecular inputs into the gate which is composed of inactive *Mtb*PYK and its substrates (red trapezoid). The enzyme *Mtb*PYK is activated (output) by the binding of either molecule input or both molecules cooperatively at certain concentrations. The sensitivity to one input molecule is increased as the concentration of the other input molecule increases. The *Mtb*PYK tetramers in inactive T-state, AMP-activated R-state, G6P-activated R-state and AMP/G6P-activated R-state are all shown in schematic representations. Domains are highlighted in one subunit: A domain in green, B domain in grey, C domain in blue. Secondary structures and residues, that undergoes significant movements from T-state to activated R-state, are shown as cartons and sticks. AMP loops are indicated as red lines. A logic gate table is also shown on top. High = high concentration; Low = low concentration. **c** A three-dimensional (3D) graph shows the relation of activator concentrations and *Mtb*PYK activity in vitro. ‘%*V*_max_’ is expressed as the percentage of maximum velocity in the presence of saturating activators. The activities were measured in vitro using purified *Mtb*PYK in the presence of 4 mM ADP and 0.2 mM PEP. **d** Thermal shift assay results for five *Mtb*PYK complexes. All data are mean ± SEM for two independent experiments done in duplicate

Thus, the displacements of helices Cα1 and Cα4 resulting in allosteric-site reshaping are not substantially regulated by rigid-body rotation, but instead are correlated with the presence of AMP and G6P. This unique ‘synergistic allostery’ suggests that the binding of either activator is able to initiate the movements of both Cα1 and Cα4 to a certain extent and consequently reshape the allosteric sites to favour binding of the other activator. The synergy of AMP and G6P shown in structural rearrangements agrees with the findings in enzyme kinetics where the presence of one activator promotes the activation efficiency of the other (Table 1). Furthermore, the binding of activators shows a remarkable increase in the thermal stability of R-state *Mtb*PYK with a Δ*T*_m_ value of 4–9 °C (Fig. 6d; Supplementary Fig. 3), suggesting that allosteric-site reshaping enhances the stability of the R-state conformation.

It is noteworthy that the helix bundle Aα6′–Aα6 that is located at the A–A interface exhibits a ~6° rotation around the pivot near Asn268 during T- and R-state inter-conversion. No further movement is observed between R-state structures of different ligated states, suggesting that the conformational rearrangement of Aα6′–Aα6 only correlates with the concerted rocking motions, and is required in any R-state structure. The movement of Aα6′-Aα6 plays a role in forming an ‘active’ catalytic site for the binding of substrate and its cofactor (Fig. 5), in agreement with our previous findings in *Trypanosoma brucei* PYK, where we showed the reorientations of side chains of active-site residues for substrate binding^27^. In addition, another pair of A–A interface helices Aα7′ and Aα7, which display modest movement shown in the heat-map, form a H-bond across the A–A interface with the adjacent helix pair Aα6′ and Aα6 via the side-chain rotation of Arg290 (Supplementary Fig. 8). One consequence of the T and R transition is therefore to reorganise the positions of Aα6′–Aα6 and Aα7′–Aα7 to transform the catalytic site between its ‘active’ and ‘inactive’ conformations.

### A molecular logic gate senses metabolic changes in vivo

The preceding biochemical and structural results are all consistent with the idea that *Mtb*PYK functions as an ‘OR logic gate’ to tightly regulate metabolism based on integration of AMP and G6P levels during the large metabolic shifts that occur during the stress of infection. The ‘rock-and-lock’ model for trypanosome PYK allostery^26^ and the ‘dock-rock-lock’ model for human M2PYK allostery^19^ are based largely on structural evidence. Until now, a lack of bacterial PYK structures with native effectors bound precluded any comparisons between prokaryotic and eukaryotic enzymes. The *Mtb*PYK structures presented here reveal atomic-level details of a unique AMP/G6P synergism controlling PYK allostery. We propose a three-step ‘rock-shape-lock’ model to describe the synergistic allostery of *Mtb*PYK: (1) Subunits of the *Mtb*PYK tetramer rock 9° simultaneously from the T-state to the R-state (Supplementary Fig. 8a, b) in response to substrate binding, movements that are coupled with interface-loop interplay and catalytic-site formation via the movement of helices Aα6′ and Aα6. (2) AMP and G6P binding reshapes the allosteric sites by local conformational changes (Supplementary Fig. 8c–e), with the two allosteric pathways working independently or in positive cooperativity. (3) A network of interactions within and between allosteric sites is then established, which ‘double locks’ the PYK tetramer in the most catalytically efficient active state termed the ‘synergistically activated R-state’ (Supplementary Fig. 8c).

This dynamic model is consistent with the idea of an OR logic gate that enables *Mtb*PYK to sense changes in both AMP and G6P levels and appropriately modulate enzyme activity during stress-induced metabolic shifts. This logic gate regulatory activity of PYK can be understood in the context of the metabolic shift that occurs during mycobacterial nutrient deprivation mimicking the tubercular granuloma. *M. bovis* BCG is a well-established experimental surrogate of Mtb for studying metabolism. In particular, the PYK protein sequence is identical for the two. Here we used LC-MS to quantify changes in the level of glycolytic intermediates in *M. bovis* BCG subjected to nutrient deprivation. As shown in Fig. 6a, starvation caused significant reductions (up to 30-fold) of G6P compared with logarithmic growth, which should lead to downregulation of PYK. In contrast, AMP levels increased (up to 4-fold) at 4 and 10 days of starvation (S4 and S10 in Fig. 6). This should upregulate PYK activity early in nutrient deprivation. Additionally, the PYK substrates ADP and PEP accumulate at S4 and S10, where PEP is a toxic glycolytic intermediate that inhibits isocitrate dehydrogenase in the TCA cycle^9^. These shifts in PYK activity as a function of AMP and G6P levels thus satisfy the criteria for an OR logic gate (inset table in Fig. 6b) that enables PYK to sense the stress-induced reduction of G6P and increase of AMP (Fig. 6a), and to continually keep PYK primed to facilitate potential carbon co-catabolism and prevent further accumulation of toxic metabolites during the stress-induced metabolic shifts (output in Fig. 6b).

## Discussion

This flexibility of CCM is essential for mycobacterial physiology and pathogenicity, particularly under the stresses of infection^2^. Allosteric mechanisms of key CCM enzymes enable bacteria to efficiently sense the changes in metabolite levels and react immediately by allosterically regulating mechanisms to maintain homoeostasis and defence against environmental threats^5^. The results from our biochemical, structural and metabolic studies uncover a unique ‘rock-shape-lock’ allostery, which endows PYK as a molecular OR logic gate to simultaneously sense the changes of AMP and G6P levels in a synergistic manner (Fig. 6a, b). This OR gate uses AMP (the low-energy-state signal) and G6P (essential for *M. tuberculosis* pathogenesis^29, 30^) as molecular inputs to promote PYK activity (output), where either input at a certain concentration is able to fully activate the enzyme activity for the needs of the bacterium (Fig. 6c). Our findings also shed light on the importance of enzyme evolution providing *M. tuberculosis* with metabolic flexibility and the ability to adapt to challenging environmental changes. The conservation of key residues in AMP and G6P binding suggests that the above allosteric model is likely to apply to other bacterial PYKs. Importantly, our results provide both structural foundation and allosteric mechanism for designing *Mtb*PYK inhibitors that could potentially provide a fast-killing therapy in combination with respiratory ATPase inhibitors like bedaquiline^8^ to completely block ATP production.

## Methods

### Cloning and protein production

A series of gene codon-optimisation procedures from Bio Basic Inc. were applied to improve the efficiency of soluble expression of *Mtb*PYK in *E. coli* (Supplementary Table 6). The codon-optimised gene was synthesised and cloned into a pYUB28b-TEV vector by Bio Basic Inc. The vector pYUB28b-TEV is modified from pYUB28b (Addgene #37277) that was provided by Dr. Ghader Bashiri from The University of Auckland. The verified construct (pYUB28b-TEV_*Mtb*PYK) allows the expression of N-terminal 6xhistidine-tagged *Mtb*PYK with subsequent tag removal by TEV protease digestion.

*Mtb*PYK was overexpressed in *E. coli* BL21(DE3) cells (Novagen, Merck Millipore) and purified as described previously^27^ with some modifications. Briefly, 10 ml of overnight culture was inoculated in 500 ml of 2xTY medium with 50 μg ml^−1^ hygromycin B and grown to OD_600_ = 0.6–0.8 at 37 °C. *Mtb*PYK protein overexpression was induced by 0.5 mM IPTG after a 30 min incubation at 4 °C. The cells were further cultured for another 24 h at 16 °C before harvesting.

Cell pellets were lysed in ice-cold buffer (50 mM triethanolamine-HCl (TEA) pH 7.2, 300 mM KCl, 20 mM imidazole, 10% glycerol) using a Constant Systems Cell Disruptor (Panda) (set to 850 bar). The soluble fraction was obtained by centrifugation at 39,191 × *g* for 45 min. The supernatant was filtered through a 0.22 μm syringe filter then applied onto an ÄKTA system fitted with 1 ml HisTrap HP IMAC column (GE Healthcare) (prepacked with Ni Sepharose). The column was maintained at a constant flow rate of 1.0 ml min^−1^ throughout the whole-purification process. Following loading of the clarified lysate, the column was washed with 20 column volumes of cell-lysis buffer. The target enzyme *Mtb*PYK was eluted from the column by applying 100% elution buffer (50 mM TEA pH 7.2, 300 mM KCl, 500 mM imidazole, 10% glycerol) and digested with TEV protease at 4 °C for 12 h to remove the 6xhistidine tag. The digested sample was dialysed against lysis buffer and loaded onto a HisTrap HP IMAC column (pre-equilibrated in cell-lysis buffer). The untagged enzyme *Mtb*PYK was collected from the flow-through fractions and concentrated to 5 ml using a Vivaspin column (GE Healthcare, 100 kDa cutoff).

Concentrated proteins were then loaded onto a HiLoad 16/600 Superdex 200 prep grade gel-filtration column with a flow rate of 1.0 ml min^−1^ in a buffer containing 20 mM TEA pH 7.2, 50 mM KCl, 10 mM MgCl_2_, 20% glycerol. *Mtb*PYK proteins eluted in a peak at ~63 ml and were further analysed on SDS–PAGE. Pure proteins (>95% purity) were concentrated to at least 20 mg ml^−1^ and were stored at −80 °C. The protein concentration was determined by measuring the absorbance at 280 nm with an extinction coefficient for *Mtb*PYK of 25,440 M^−1^ cm^−1^.

### Enzyme activity assay and kinetic studies

The activity and kinetics of *Mtb*PYK were determined as described for *T. brucei* PYK previously^27^ with some modifications. Briefly, PYK activity was measured by following the decrease in NADH absorbance at 340 nm using a BioTek Synergy 4 microplate reader and the data were analysed by the software Graphpad Prism 7. The assay was performed at 25 °C in 100 μl reaction mixtures consisting of 1x assay buffer (50 mM TEA pH 7.2, 100 mM KCl, 10 mM MgCl_2_), 0.5 mM NADH, 3.2 U LDH and 1 μg ml^−1^ *Mtb*PYK. The specific activity of *Mtb*PYK was calculated from the corresponding kinetic curve. One activity unit is the reaction of 1 μmol substrate per minute under the condition used in this assay. The turnover value (*k*_cat_) of *Mtb*PYK was calculated from the specific activity divided by the subunit molar mass of 50,950.4 g mol^−1^.

Enzyme kinetics with respect to ADP were studied with saturated PEP (8 mM) and at variable concentrations of ADP (from 0 to 4 mM). Enzyme kinetics with respect to PEP were studied with saturated ADP (4 mM) and at variable concentrations of PEP (from 0 to 8 mM) in the presence or absence of 1 mM activator AMP or G6P, or 2 mM inhibitor ATP. The combinations of effectors were also studied: 1 mM AMP plus 1 mM G6P with or without 2 mM ATP. The hyperbolic kinetics with respect to ADP and sigmoidal kinetics with respect to PEP were analysed using Graphpad Prism 7, respectively.

Enzyme kinetics with respect to the synergistic activators AMP and G6P were studied at 4 mM ADP, 0.2 mM PEP and variable concentrations of AMP and G6P. The synergistic activation was studied by adding G6P or AMP in AMP titration or G6P titration. The synergistic activation by AMP was studied in the presence of G6P (25 or 50 µM) and variable concentrations of AMP (from 0 to 3.2 mM). The synergistic activation by G6P was similarly studied in the presence of AMP (12.5 or 25 µM) and variable concentrations of G6P (from 0 to 3.2 mM). The enzyme kinetics with respect to allosteric effectors were analysed with Graphpad Prism 7 using an allosteric sigmoidal model. All kinetic results are summarised in Table 1. A 3D bar illustration was plotted by MATLAB showing the relation of effector concentrations and *Mtb*PYK enzyme activity, where *x*/*y*-axis represents the effector concentration and the height (*z*-axis) is the percentage (%) of enzyme’s maximum rate (*V*_max_).

### Thermal shift assay

The thermal stability analysis of *Mtb*PYK was determined as described for *T. brucei* PYK previously^27^. Briefly, the assay was performed in a 96-well PCR plate (Bio-Rad) with 50 μl per reaction containing 5× SYPRO Orange dye (Invitrogen), 4 μM *Mtb*PYK enzyme and 10 mM of the test ligand(s) (PEP, oxalate, pyruvate, ATP, AMP, G6P, F16BP, R5P, PO_4_). The assay buffer (50 mM TEA pH 7.2, 100 mM KCl, 10 mM MgCl_2_) was added instead of the test ligand as a negative control. To start the experiment, the temperature was increased from 25 to 95 °C in an i-Cycler iQ5 real-time PCR system (Bio-Rad). The thermal stability curve was analysed using the Bio-Rad iQ5 software, then the temperature midpoint *T*_m_ for the protein-unfolding transition was calculated.

### Crystallization and data collection

The crystallisation experiments were performed by the vapour-diffusion method using the hanging-drop technique at 4 °C. The drops were equilibrated against a reservoir filled with 1 ml well solution. To crystallise *Mtb*PYK in the inactive state (T-state), drops were formed by mixing 1.5 μl well solution with 1.0 μl protein solution (20 mg ml^−1^). To co-crystallise the enzyme with the PEP analogue oxalate (OX), product ATP or synergistic activators G6P and AMP, 1.0 μl protein solution was mixed with 0.5 μl ligand solution (20 mM) and incubated at room temperature for 1–2 min. Then 1.5 μl well solution was added to the mixture for crystallisation. Oxalate is a structural analogue of the enolate form of pyruvate and has been generally used in crystallisation conditions to stabilise PYK in the active R-state^26, 27^. The well solution consisted of 6–20% PEG 8000, 10-20% glycerol, 50 mM TEA buffer pH 7.2, 100 mM KCl, 50 mM MgCl_2_. Crystals of T-state *Mtb*PYK, R-states *Mtb*PYK-OX, *Mtb*PYK-OX/ATP/AMP and *Mtb*PYK-OX/AMP/G6P were grown in conditions consisting of 11–16% PEG 8000 plus 10–20% glycerol. Although ATP was also added to the crystallisation conditions of the crystals *Mtb*PYK-OX and *Mtb*PYK-OX/AMP/G6P, electron density of ATP was not discovered in either structure. Crystals of *Mtb*PYK-OX/G6P and *Mtb*PYK-OX/AMP/G6P (different from the co-crystallised one) were obtained by soaking *Mtb*PYK-OX crystals with 5 mM G6P and 5 mM AMP/G6P mixture, respectively. The soaking solution contained 5 mM for each ligand, 20% PEG 8000 and 20% glycerol.

X-ray intensity data for crystals of T-state *Mtb*PYK and *Mtb*PYK-OX/ATP/AMP were collected at the Swiss Light source (SLS, Switzerland) while the intensity data for crystals *Mtb*PYK-OX, *Mtb*PYK-OX/G6P, *Mtb*PYK-OX/AMP/G6P and *Mtb*PYK-OX/AMP/G6P (soak) were collected at the Australian Synchrotron (Australia). Each data set was from a single crystal flash-cooled in liquid nitrogen at 100 K. Data were then processed with MOSFLM^31^ and scaled with AIMLESS^32, 33^. The data-collection and processing statistics are summarised in Table 2.

### Structure determination

The T-state *Mtb*PYK structure was solved by molecular replacement using the program Phaser^34^. The initial search model (*Mtb*PYK monomer) for the molecular-replacement experiment was obtained from the I-TASSER server^35^. The structure was manually adjusted using Coot^36^ followed by several cycles of restrained refinement in REFMAC^37^. Where appropriate, water molecules and ligands were added to the structure and TLS refinement was applied at later stage of refinement. The mobile B-domain of chain C has poor density that could not be improved after refinement. Thus, this B-domain (residues 72–164) was deleted from the final refined structure.

The diffraction data sets of the R-state *Mtb*PYK structures [*Mtb*PYK-OX, *Mtb*PYK-OX/ATP/AMP, *Mtb*PYK-OX/G6P, *Mtb*PYK-OX/AMP/G6P and *Mtb*PYK-OX/AMP/G6P (soak)] were scaled to the space group of P6_4_22 with reasonable statistics assessed by the program AIMLESS^32, 33^. The refined structure of the T-state *Mtb*PYK monomer was used as the search model for molecular replacement by Phaser^34^. After refinement using REFMAC^37^ and manual model building in Coot^36^, the *R*/*R*_free_ remained high (both values >0.40 for data sets at 1.9–2.8 Å resolution). The L-test results indicated merohedral twinning had occurred in data sets, although there is no twin law in the space group of P6_4_22. The data sets were then scaled to all possible space groups in lower symmetry with possible twin laws. The results of molecular replacement and refinement indicate the data sets scaled in P3_1_ have the most reasonable *R*/*R*_free_ values (0.15–0.20) and density qualities. The solved structures determined in space group P3_1_ were further refined by following the procedures used for the T-state structure refinement.

The quality of the structures was assessed using the MOLPROBITY server^38^, and the figures were generated using PyMOL^39^. The data processing and refinement statistics are summarised in Table 2. The structure factors and coordinates for T-state *Mtb*PYK, *Mtb*PYK-OX, *Mtb*PYK-OX/ATP/AMP, *Mtb*PYK-OX/G6P, *Mtb*PYK-OX/AMP/G6P and *Mtb*PYK-OX/AMP/G6P (soak) have been deposited in the RCSB Protein Data Bank as PDB entries 5WRP, 5WS8, 5WS9, 5WSA, 5WSB and 5WSC, respectively.

### Structure analysis

The program Superpose^40^ in the CCP4^41^ suite was used to calculate the allosteric rigid-body rotations from the superposition of T-state and R-state tetramers as described previously^26^. Both RMS differences and rotation matrices were calculated in the superposition process^26^.

The equivalent Cα atom distances of superposed structures are analysed by heat-map analysis to highlight the conformational changes within structures. AC-cores (A and C domains, residues 1–70 and 168–472) from each R-state *Mtb*PYK structure were superposed (Cα superposition) onto the AC-core of the T-state structure using program Superpose^40^. Values of equivalent Cα distances (Dist.) from each pair of superposition were used to generate heat maps by GraphPad Prism 7. The increase of distance is represented as a blue to red gradient.

### Molecular simulations and structural network analysis

Three *Mtb*PYK crystal structures with OX/G6P, OX/AMP, OX/AMP/G6P bound, were prepared using Visual Molecular Dynamics (VMD)^42^. Each tetrameric structure was solvated in a sufficiently large water box with salinity set to 150 mM NaCl. The systems, typically involving 220,000 atoms, were then subjected to all-atom MD simulations for 2 ns with positional restraints to the backbone atoms and a further 150 ns without any restraints. The simulations were performed using NAMD 2.10^43^ assuming the CHARMM36 force field for the protein^44^ and assuming the TIP3P model for water molecules^45^. The CHARMM parameters for oxalate, AMP and G6P were obtained from cgenff^46, 47^. Network analysis was performed to investigate the dynamic coupling between oxalate and AMP/G6P. We calculated 1000 highly correlated paths of residues between ligand sites of interest for every *Mtb*PYK monomer by employing the weighted implementation of suboptimal paths (WISP) algorithm^28, 48^. Therefore, a total of 4000 suboptimal paths were collected for the *Mtb*PYK tetramer. To perform community network analysis^49^, a weighted network was first constructed where each residue represents a node and the weight of the connection between nodes represents their respective correlation values. Communities were then identified such that each community contains a group of highly correlated residues^50^. Note that the simulation has converged after 20 ns (Supplementary Fig. 10) and all analyses were done on the last 100 ns of the MD simulation trajectory with 20 ps interval.

### *Mycobacterium bovis* BCG culture and nutrient starvation

Isogenic freezer stocks of *Mycobacterium bovis* bacille Calmette Guérin (BCG) Pasteur strain 1172P2 were used for starter cultures in 50 ml conical tubes, which were subsequently passaged into roller bottles with supplemented 7H9 medium^51^. To obtain nutrient-deprived cultures, BCG were grown to OD_600_ of 0.8–1.0 and washed twice in PBS (pH 7.4) containing 137 mM NaCl, 2.7 mM KCl, 10 mM NaH_2_PO_4_, 1.8 mM KH_2_PO_4_, then re-suspended in PBS with 0.05% Tyloxapol to an OD_600_ of ~1.0 in roller bottles for up to 22 days. At 4, 10 and 22 d post-nutrient deprivation (termed S4, S10 and S22, respectively), samples from 4 independent cultures were taken for metabolic assessment. The remaining samples at S22 were re-inoculated into nutrient replete 7H9 at a starting OD_600_ of 0.1. The metabolites in these cultures (termed R6) are assessed 6 d post inoculation. CFUs for BCG were determined from 10-fold serial dilution plating on 7H10 agar at 37 °C for 3 w.

### Metabolite extraction and targeted metabolomics

Metabolite extraction followed the published report^52^. Briefly, cell cultures were harvested at given time points, rapidly quenched and spun down. Cell pellets were resuspended in acetonitrile:methanol:water (2:2:1) and lysed mechanically with 0.1-mm silica beads by using Qiagen TissuelyserII. The lysates were collected and evaporated to dryness in a vacuum evaporator, and the dry extracts were redissolved in 100 µl of 98:2 water/methanol for liquid chromatography-mass spectrometry (LC-MS) analysis.

The targeted LC-MS/MS analysis was performed with Agilent 1290 ultrahigh pressure liquid chromatography system coupled to a 6460 Triple Quadrupole mass spectrometer equipped with a dual-spray electrospray ionization source with Jet Stream^TM^ (Agilent Technologies, Santa Clara, CA). Chromatographic separation was achieved by using Phenomenex (Torrance, CA) Rezex^TM^ ROA-Organic Acid H + (8%) column (2.1 × 100 mm, 3 µm) and the compounds were eluted at 40 °C with an isocratic flow rate of 0.3 ml min^−1^ of 0.1% formic acid in water. The auto-sampler was cooled at 4 °C and an injection volume of 5 μl was used. Electrospray ionisation was performed in negative ion mode with the following source parameters: drying gas temperature 300 °C with a flow of 10 l min^−1^, nebulizer gas pressure 40 psi, sheath gas temperature 350 °C with a flow of 11 l min^−1^, capillary voltage 3000 V and nozzle voltage 500 V. Compounds were quantified in multiple reaction monitoring (MRM) mode with the following transitions: *m/z* 426 > 124 and *m/z* 426 > 79 for adenosine diphosphate (ADP), *m/z* 259 > 199 and *m/z* 259 > 138.9 for glucose 6-phosphate (G6P), *m/z* 87 > 43.1 and *m/z* 87 > 32.1 for pyruvate, *m/z* 167 > 79 and *m/z* 167 > 63 for phosphoenolpyruvic acid (PEP), *m/z* 229 > 169.1 and *m/z* 229 > 138.9 for ribose 5-phosphate (R6P), *m/z* 339 > 96.9 and *m/z* 339 > 79 for fructose 1,6-bisphosphate (F16BP). Adenosine monophosphate (AMP) was analysed using Agilent 1290 ultrahigh pressure liquid chromatography system equipped with a 6520 QTOF mass spectrometer equipped with a dual-spray electrospray ionization source (Agilent Technologies, Santa Clara, CA). The column used for the separation was an Agilent rapid resolution HT Zorbax SB-C18 (2.1×100 mm, 1.8 mm). The oven temperature was set at 45 °C and the flow rate was set at 0.4 ml min^−1^. The gradient mobile phase consisted of 0.1% formic acid in water (A) and 0.1% formic acid in methanol (B). Metabolites were eluted with the following gradient: 0 to 7 min, 2–70% B; 7 to 8 min, 70–100% B; 8–11 min, hold at 100% B, 11–11.1 min, 100-2% B. The auto-sampler was cooled at 4 °C and a 5 μl injection volume was used. Mass spectrometry was performed at positive ion electrospray ionization mode and the mass data were collected between *m/z* 100 and 600 Da at a scan rate of 2 spectra per second. The ion spray voltage and the heated capillary temperature was set at 4000 V and 350 °C, respectively The drying gas and nebulizer nitrogen gas flow rates were set to 12.0 l min^−1^ and 50 psi, respectively. Two reference masses were continuously infused to the system to ensure mass accuracy during the run: *m/z* 121.0509 (C_5_H_4_N_4_) and *m/z* 922.0098 (C_18_H_18_O_6_N_3_P_3_F_24_). The MassHunter software (Agilent Technologies, USA) was used to collect and process the data. Residual protein content was determined to normalise samples to cell biomass (BCA protein assay kit; Thermo Scientific). The metabolic abundance data were normalised to protein concentration.

### Data availability

Coordinates and structure factors for the T-state structure *Mtb*PYK, R-state complexes *Mtb*PYK-OX, *Mtb*PYK-OX/AMP/ATP, *Mtb*PYK-OX/G6P, *Mtb*PYK-OX/AMP/G6P, *Mtb*PYK-OX/AMP/G6P (soaking method) have been deposited in the Protein Data Bank under accession codes 5WRP, 5WS8, 5WS9, 5WSA, 5WSB and 5WSC, respectively. The authors declare that the data supporting the findings of this study are available within the article and its Supplementary Information Files, or from the corresponding authors upon reasonable request.

## Electronic supplementary material

Supplementary Information

Description of Additional Supplementary Files

Supplementary Movie 1

Supplementary Movie 2

Supplementary Movie 3
