# Supplementary material for: Allosteric pyruvate kinase-based “logic gate” synergistically senses energy and sugar levels in Mycobacterium tuberculosis
Source: Nat Commun. 2017 Dec 7;8:1986. doi: 10.1038/s41467-017-02086-y (PMC5719368; doi:10.1038/s41467-017-02086-y)
Supplement: Supplementary file 1 — Supplementary Information [file 41467_2017_2086_MOESM1_ESM.pdf]

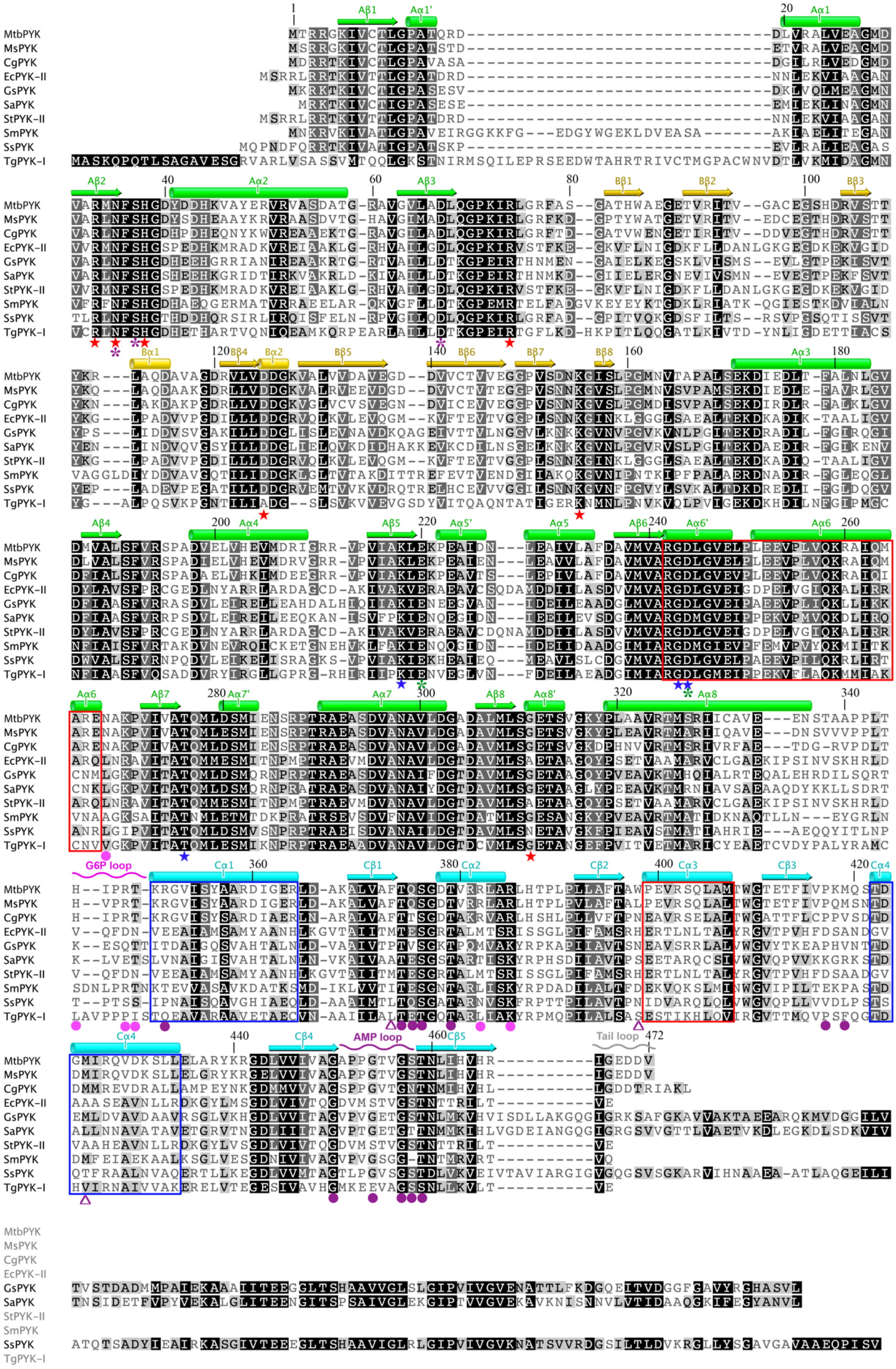
MtbPYK
MsPYK
CgPYK
EcPYK-II
GsPYK
SaPYK
StPYK-II
SmPYK
SsPYK
TgPYK-I
G6P loop
AMP loop
Tail loop

Supplementary Figure 1. Sequence alignment of pyruvate kinases. Amino acid sequences of selected bacterial pyruvate kinases were aligned by the program ClustalW2[1,2] and visualised by the software Geneious[3]. These selected pyruvate kinases are activated by at least AMP or G6P. The conservation of the residues is indicated by shading from black (identical in nine or ten sequences) to grey (conserved in seven or eight) to white (low or no conservation). Residue numbers corresponding to *Mtb*PYK are listed above the sequence. Secondary structural elements defined in the *Mtb*PYK crystal structure are shown above the sequences (only α-helices and β-strands are shown). Secondary structural elements are labelled in different colours corresponding to their domain regions: A domain (residues 1-70 & 168-336: green), B domain (residues 71-167: yellow) and C domain (residues 337-472: cyan). The AMP loop (residues 451-458), G6P loop (residues 345-349) and C-terminal tail loop (residues 467-472) are indicated by wave lines above the sequence in purple, magenta and grey, respectively. The α helixes (Aα6'-Aα6 and Cα3) that bridge the allosteric site and active site are highlighted in red boxes, while Cα1 and Cα4 that undergo significant structural rearrangements in AMP/G6P synergistic activation are indicated in blue boxes. In *Mtb*PYK, the amino acids involved in magnesium metal ion binding (green asterisks), potassium metal ion binding (purple asterisks), substrate analogue oxalate binding (blue stars), product ATP binding (red stars), synergistic activator AMP binding (purple circles and triangles) and synergistic activator G6P binding (magenta circles) are indicated below the aligned sequences. Three residues that form a π-stacking core with the adenosine ring of AMP are indicated using purple triangles. *Mtb, Mycobacterium tuberculosis* (UniProtKB: P9WKE5); *Ms, Mycobacterium smegmatis* (UniProtKB: A0QXA3); *Cg, Corynebacterium glutamicum* (UniProtKB: Q46078); *Ec, Escherichia coli* (UniProtKB: P21599); *Gs, Geobacillus stearothermophilus* (UniProtKB: Q02499); *Sa, Staphylococcus aureus* (UniProtKB: Q6GG09); *St, Salmonella typhimurium* (UniProtKB: Q8ZNW0); *Sm, Streptococcus mutans* (UniProtKB: Q8DTX7); *Ss, Synechococcus sp.* (UniProtKB: A0A0H3K2W0); *Tg, Toxoplasma gondii* (UniProtKB: Q969A2).

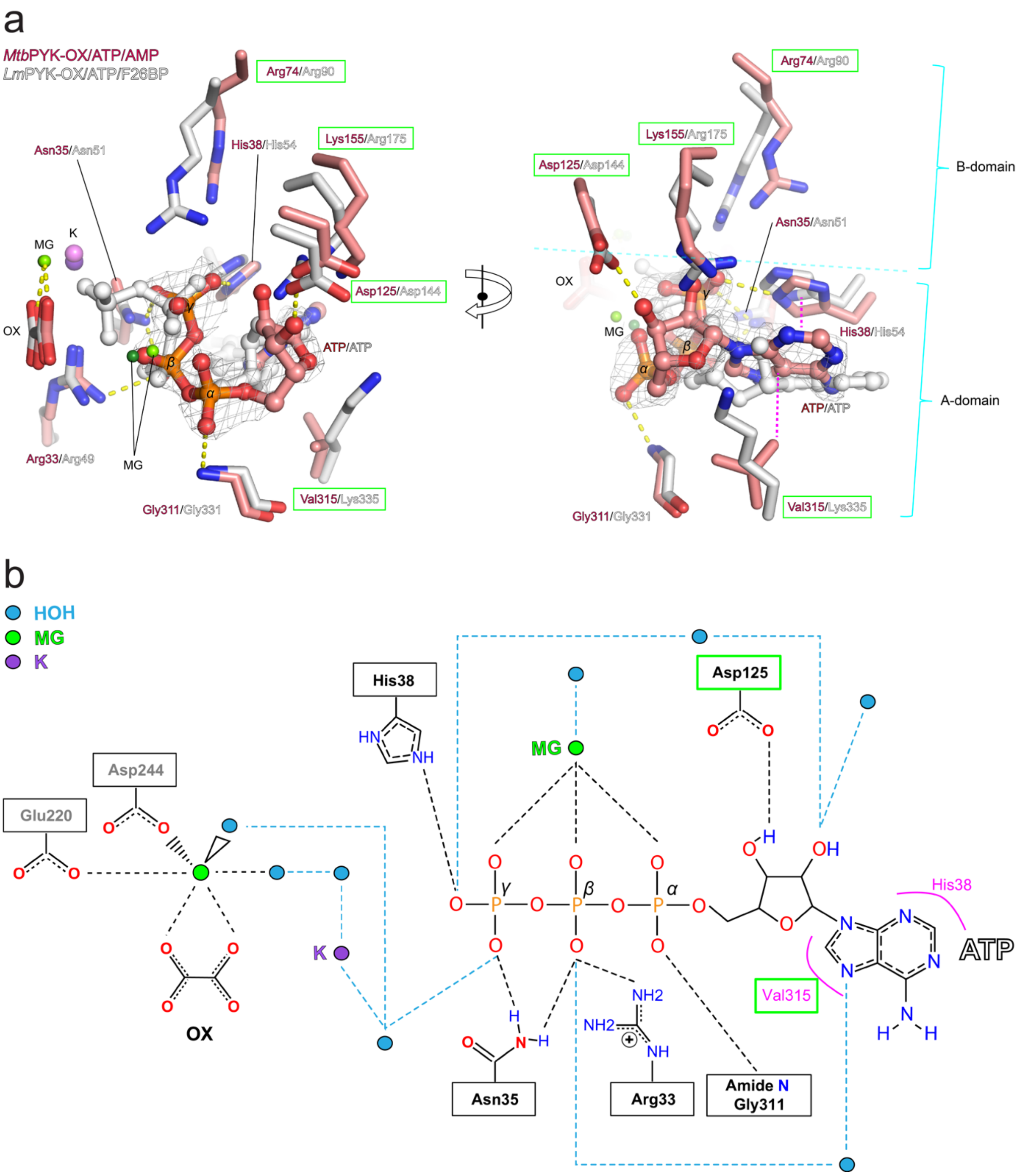

Supplementary Figure 2. Active site of *Mtb*PYK indicating the binding mode of ATP. (**a**) Two orthogonal views of superposed active sites of *Mtb*PYK-OX/ATP/AMP (chain C, salmon) and *Lm*PYK-OX/ATP/F26BP (chain C, PDB: 3HQP, grey) showing the interactions at the active site. Ligands and interacting residues are shown as sticks while ions are shown by spheres. Interactions are indicated by yellow dashed lines, and stacking interactions are shown using pink dashed lines. ATP in the *Mtb*PYK structure is shown by an unbiased *Fo-Fc* electron density map (grey) contoured at 3σ. Water molecules are removed for clarity. (**b**) A schematic representation showing the interatomic interactions at the active site of the *Mtb*PYK-OX/ATP/AMP complex. Residues forming stacking interactions with the adenine ring of ATP are indicated in pink.

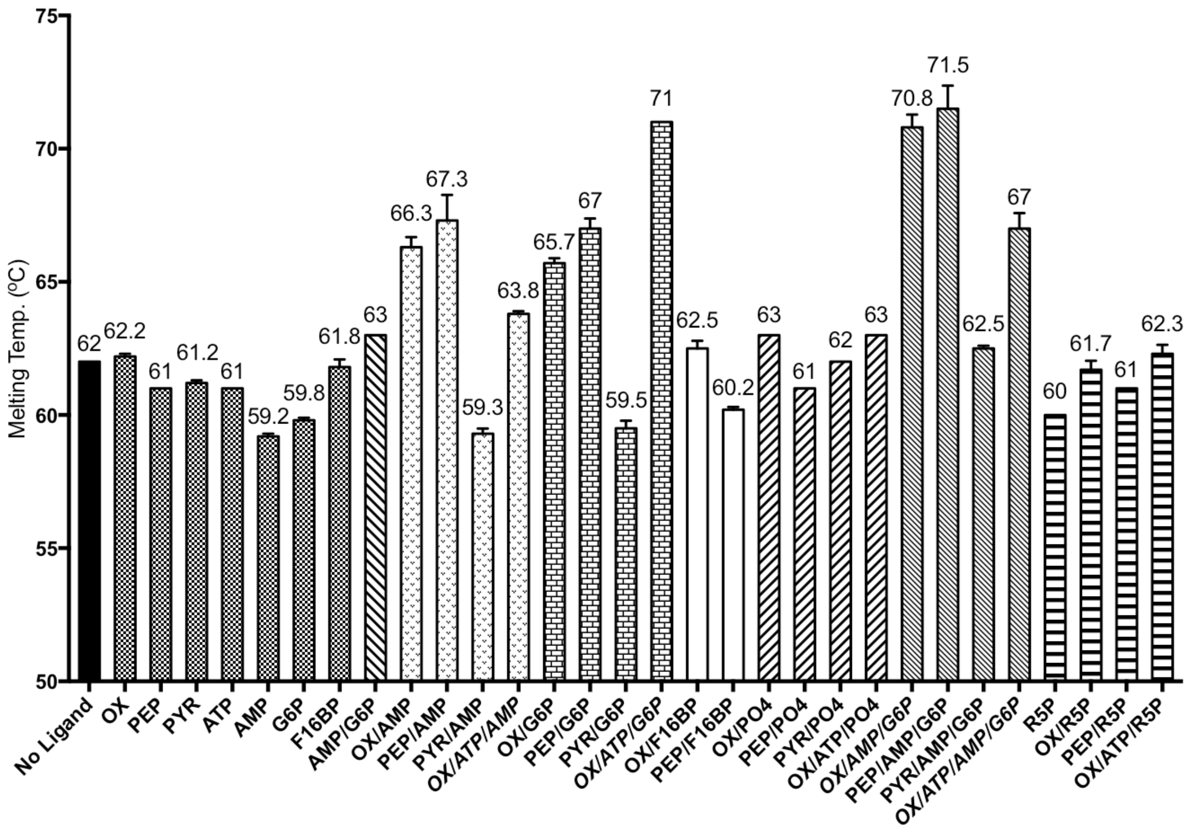

Supplementary Figure 3. Stabilisation of *Mtb*PYK by binding small molecules. Thermal shift assay results for *Mtb*PYK in the absence (black bar) and the presence of test ligands. The melting temperature T_m values (^{o}C) are shown above the bars. All data are mean ± SEM for two independent experiments done in duplicate. Different types of shading represent various groups of ligands.

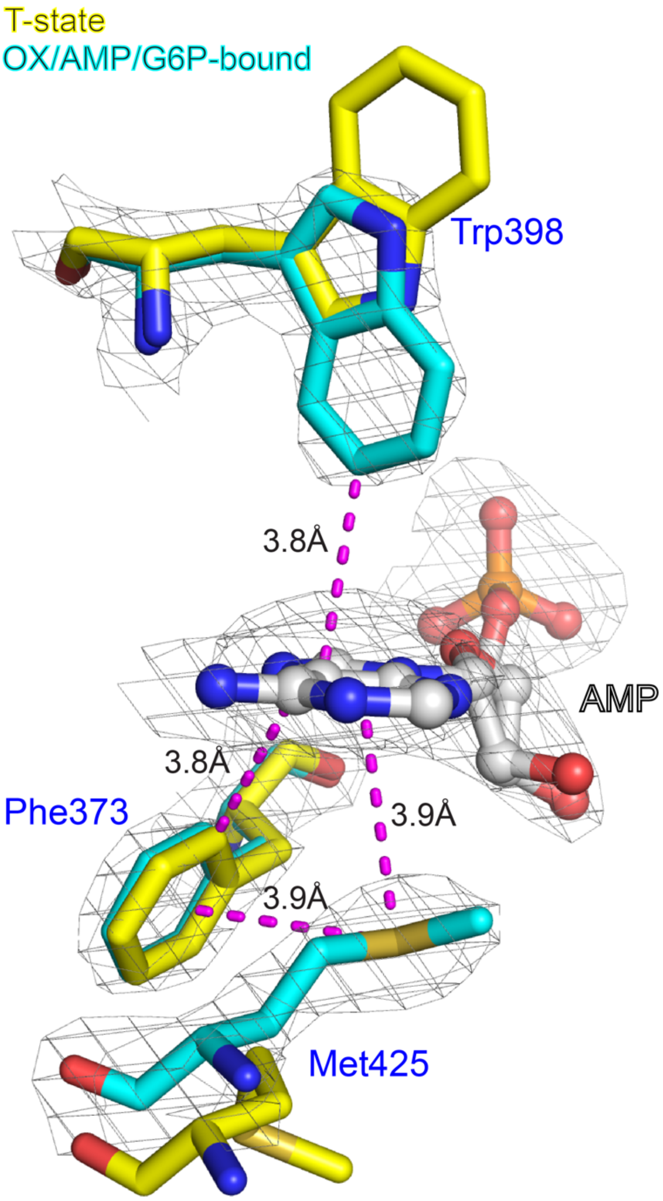

Supplementary Figure 4. Close-up view of π-stacking core at AMP binding site. Effector-site superposition of T-state *Mtb*PYK structure (yellow) and OX/AMP/G6P-bound structure (cyan) indicating the conformational changes of residues (shown as sticks) to form stacking interactions (CH–π hydrogen bonds; pink dashed lines) with the adenosine ring of AMP. The interacting distances are given in Ångstroms. Residues of the OX/AMP/G6P-bound structure and AMP are shown with unbiased *Fo-Fc* electron density maps (grey) contoured at 3.0 σ.

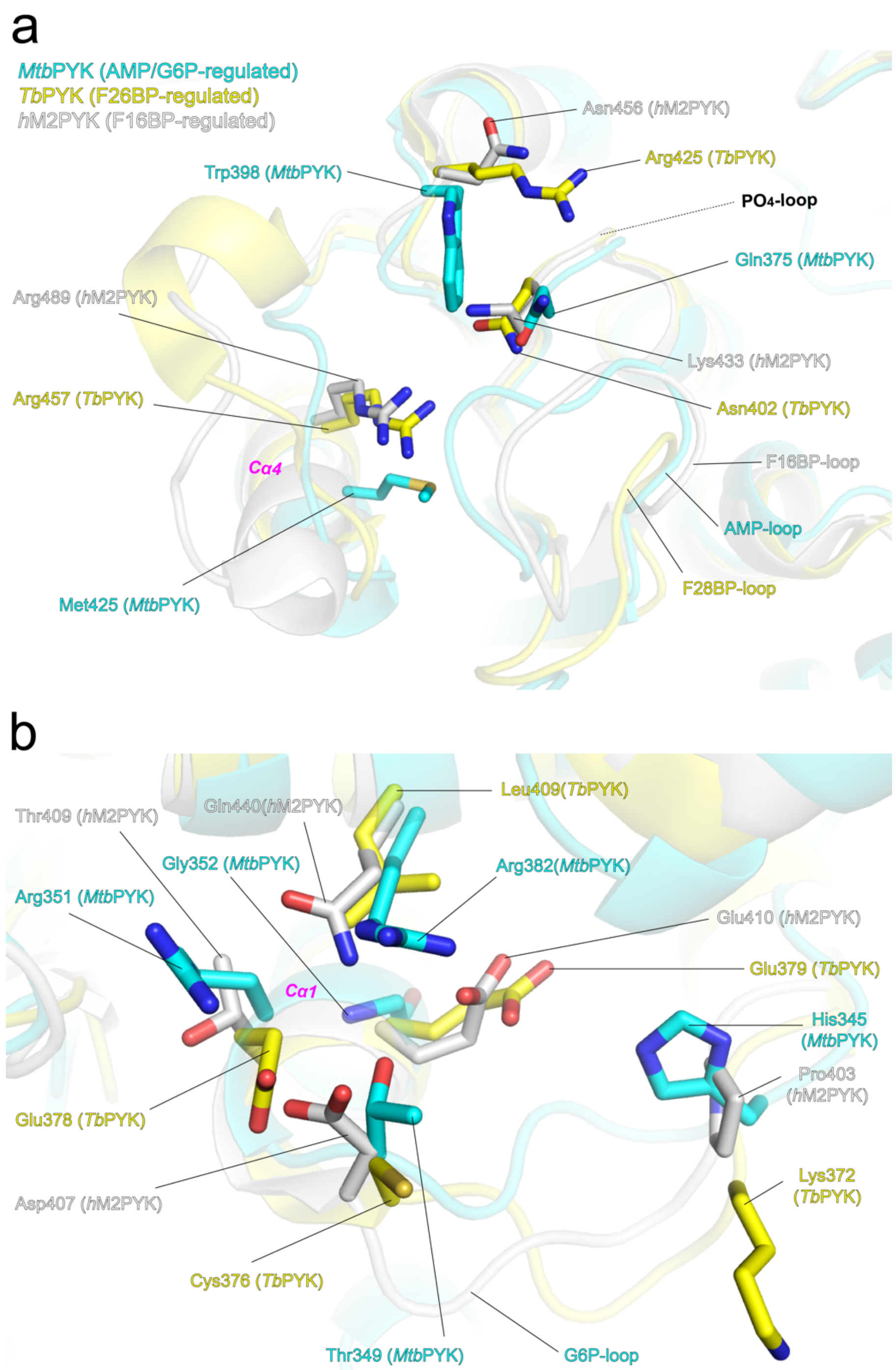

Supplementary Figure 5. Effector-site residues are diverse in PYKs that are regulated by different effectors. Close-up view of the superposed C-domains of *Mtb*PYK-AMP/G6P (cyan), *T. brucei* PYK in complex with F26BP (*Tb*PYK-F26BP in yellow; PDB ID: 4HYW) and human M2PYK-OX/ATP/F1BP (grey; PDB ID: 4FXF). The polypeptide chains are shown as cartons while essential effector-site residues are shown as sticks. Allosteric effectors are removed for clarity. (a) Canonical effector site for AMP, F26BP or F16BP binding. (b) G6P binding site in *Mtb*PYK.

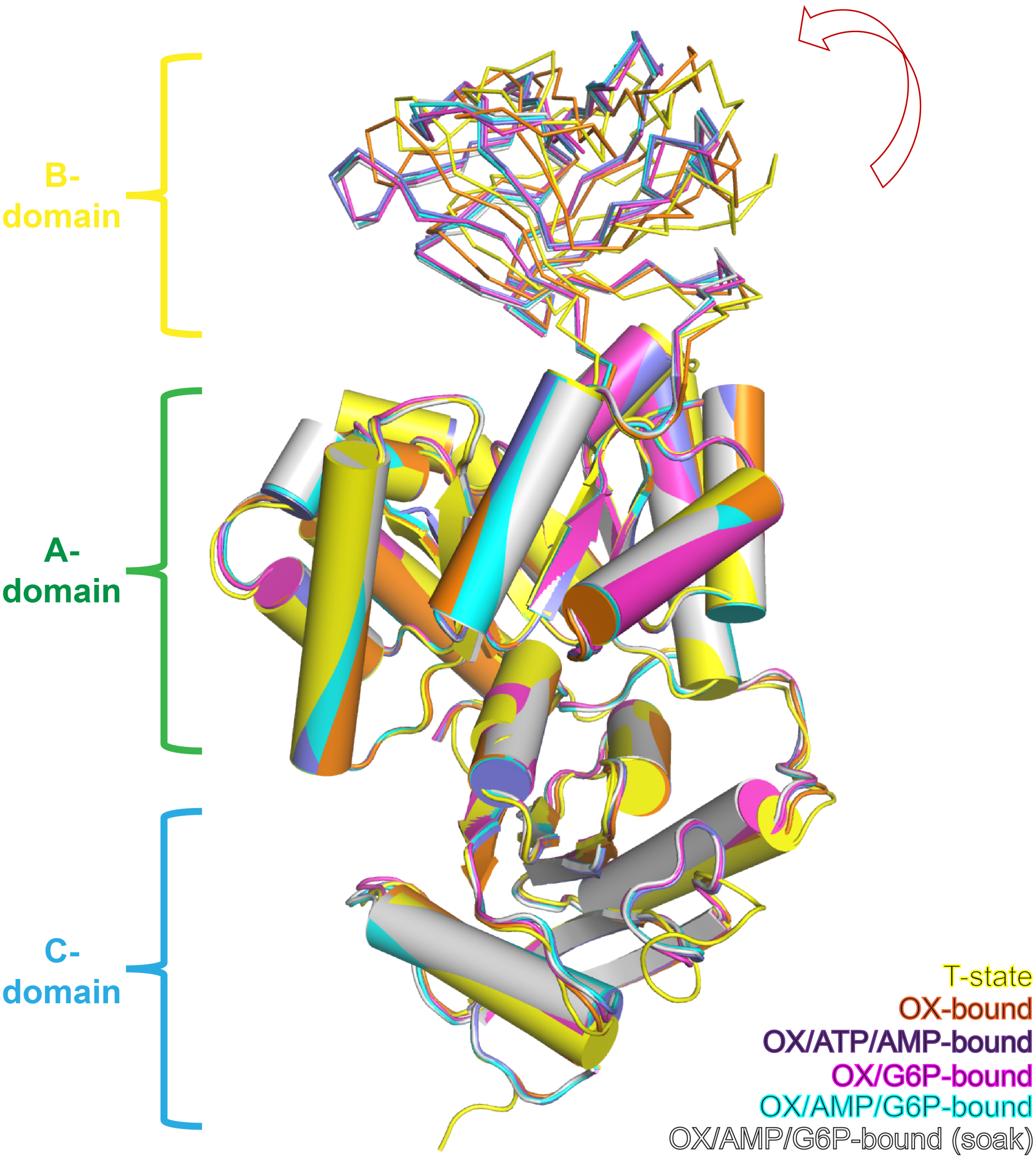

Supplementary Figure 6. B-domain motions in allosteric transitions. Subunits from six different *Mtb*PYK structures (subunit D in each structure) are superposed based on the AC-core (A and C domains) thereby showing the movements (indicated by an arrow) of the B-domains: T-state *Mtb*PYK (yellow), *Mtb*PYK-OX (orange), *Mtb*PYK-OX/ATP/AMP (purple), *Mtb*PYK-OX/G6P (magenta), *Mtb*PYK-OX/AMP/G6P (cyan), *Mtb*PYK-OX/AMP/G6P (soak) (grey). AC-cores are shown as cartoons while the B domains are represented by ribbons. The AC-cores are well superposed with an RMS fit for all Cα atoms of ~1.0 Å (**Supplementary Table 4**). The details of the B-domain motions are described in **Supplementary Table 6**.

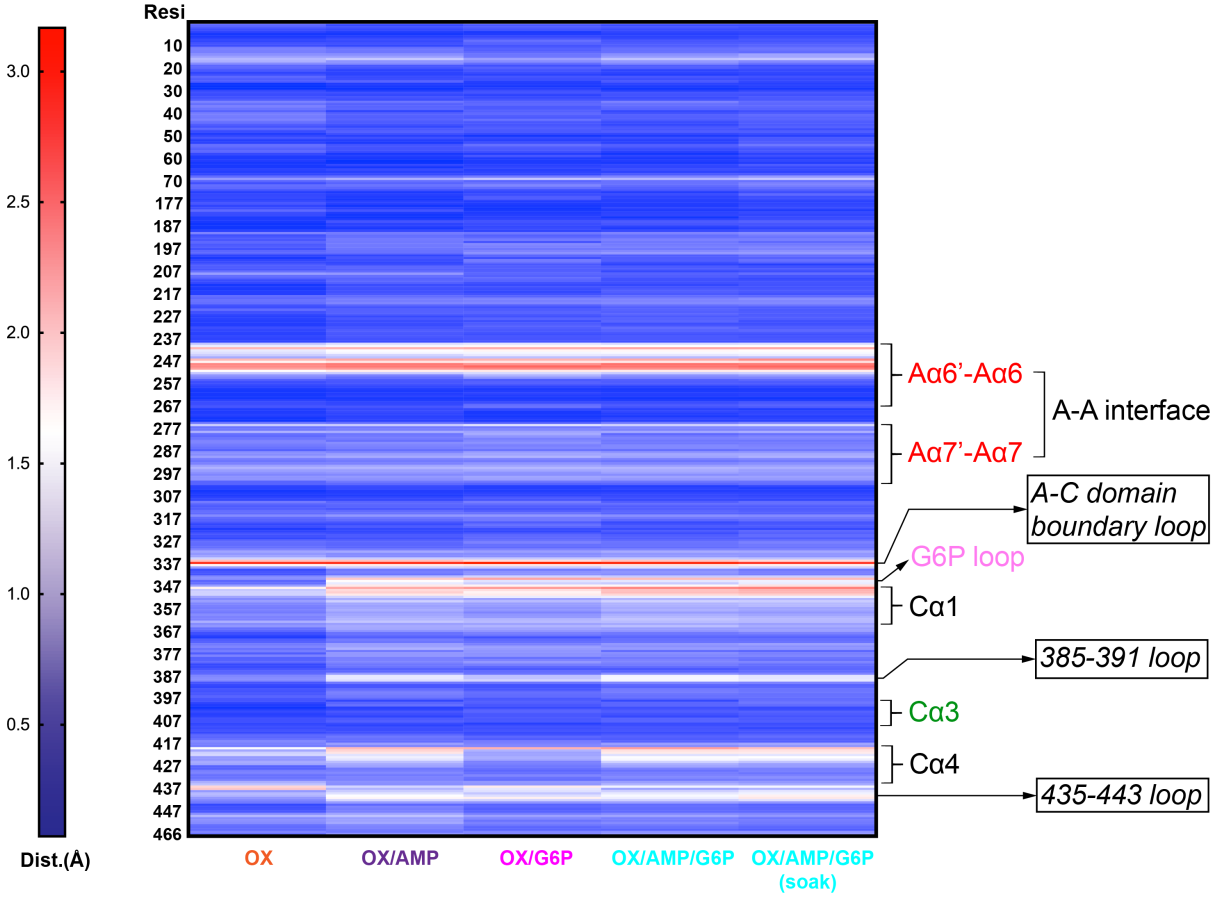

Supplementary Figure 7. The heat-map of C-α distances of various superposed R-state structures to the T-state structure showing the structural rearrangements within the AC core in different ligand-bound states. The C-α differences (AC core) of each R-state *Mtb*PYK subunit compared to the T-state subunit are represented by a gradient of blue (no movement) to red (distinct movement). The AMP loop and C-terminal tail loop have been removed in this analysis due to the significant flexibility (up to ~8-14 Å movement). The heat-map was generated by the program GraphPad Prism 7 using XYZ displacement values (distance between two corresponding atoms).

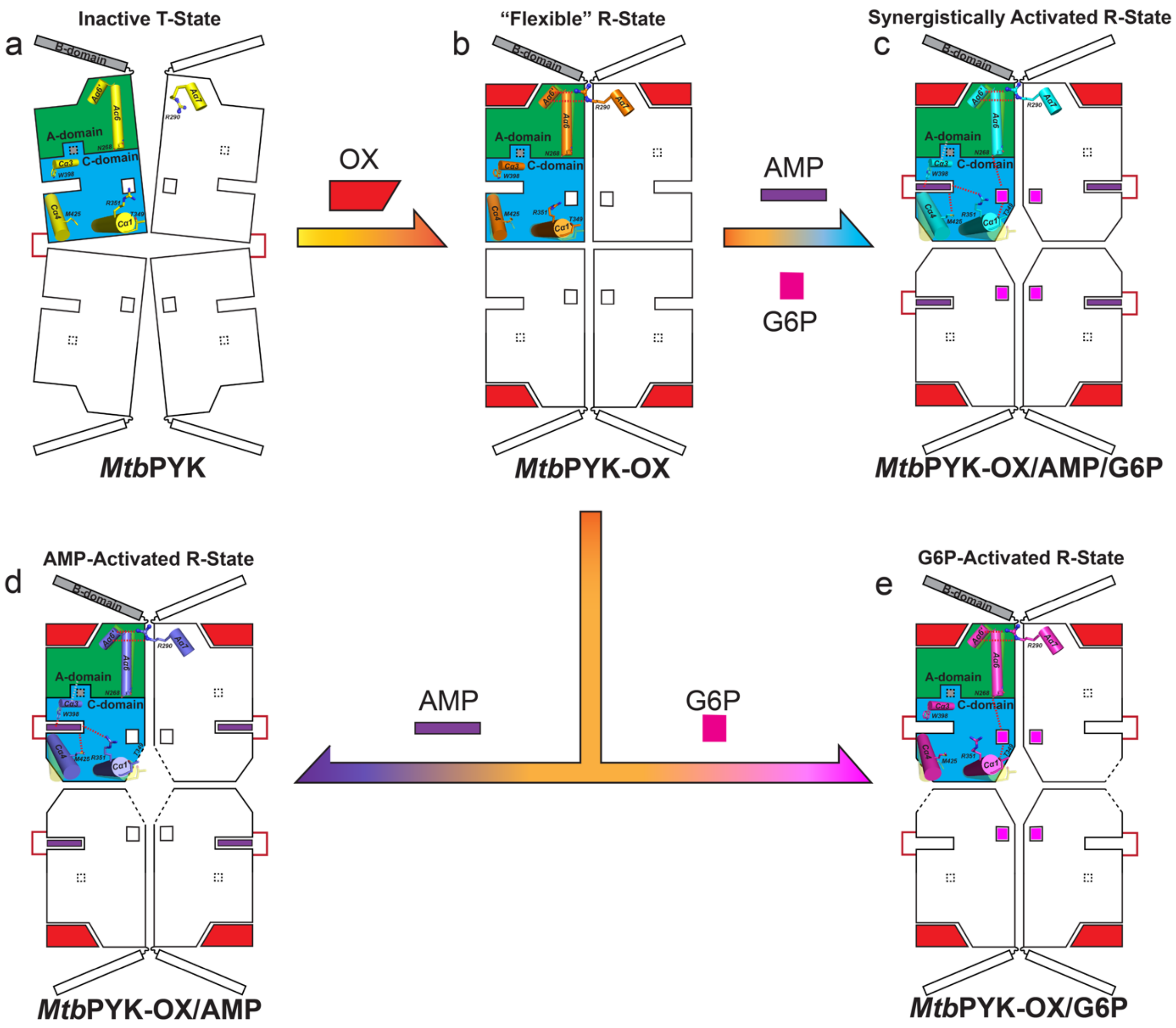

Supplementary Figure 8. The "rock-shape-lock" model of *Mtb*PYK showing structural plasticity in response to allosteric effectors. *Mtb*PYK undergoes a rigid-body rotation and allosteric-site reshaping through AMP/G6P synergistic allostery. **a**, Schematic representation of the inactive *Mtb*PYK tetramer at T-state. Domains are highlighted in one subunit: A domain in green, B domain in grey, C domain in blue. Secondary structures and residues, that undergoes significant movements from T-state to "flexible" R-state, further to synergistically activated R-state, are shown as cartons and sticks. AMP loops are indicated as red lines. **b**, Binding of oxalate (OX in red, substrate PEP analogue) to the active site induces the AC-core rotations, movement of Aα6'-Aα6, and the side-chain flip of Arg290 (Aα7) interacting with Aα6' across A-A interface. T-state elements are shown in background for comparison. In this effector-free state, AMP loop becomes disordered and loses interaction with C-terminal tail loop across C-C interface. This R-state structure without effector binding is less thermal stable (flexible) compared with effector-bound structures. **c-e**, Binding of AMP (purple) or/and G6P (magenta) induces a series of conformational changes stabilizing R-state conformation. These changes include side-chain flip of Trp398 (Cα3), AMP loop (shown as solid red line) stabilization, and movements of Cα1 and Cα4. The sugar group of G6P interacts with residue Asn268 from Aα6 that bridges allosteric and active sites. T-state elements are shown in background for comparison. The structure shown in **c** is at synergistically activated R-state which is fully stabilized by binding of both effectors.

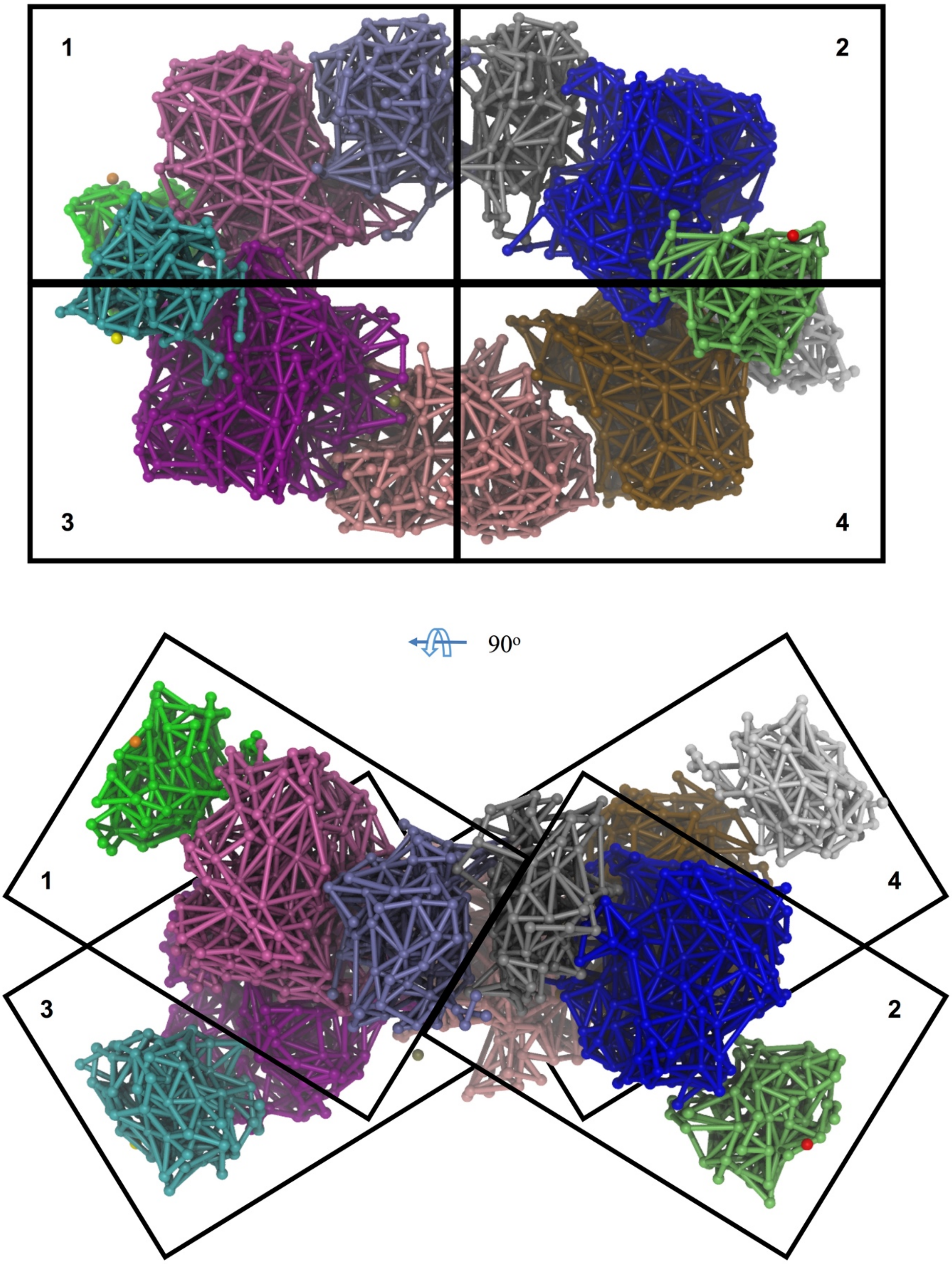

Supplementary Figure 9. Community analysis identified 12 main communities. These 12 communities correspond to the 3 domains in each monomer. The 4 monomers are numbered and boxed for clarity.

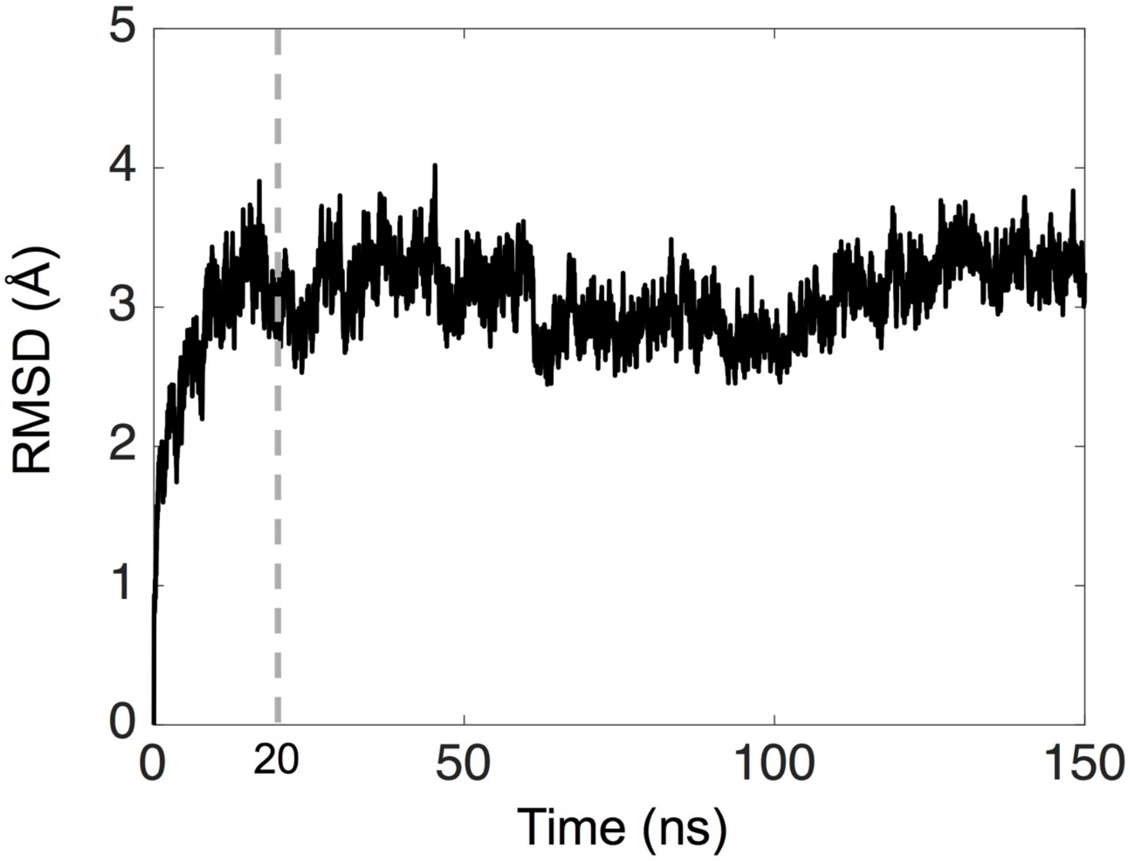

Supplementary Figure 10. The MD simulation is converged after 20 ns based on the Root Mean Square Deviation (RMSD) values. All analyses were done on simulation trajectory after 50 ns. The RMSD values are calculated on the backbone atoms.

Supplementary Table 1. Comparison of kinetic properties of PYKs.

Ligand	Modulator	Kinetic parameter	*Mtb*PYK	*Mtb*PYK	*Ms*PYK	*Cg*PYK	*Ec*PYK-II	*Gs*PYK	*Sa*PYK	*St*PYK-II	*Sm*PYK	*Ss*PYK	*Tg*PYK-I
	Reference		This work*	[4]	[5]	[6]	[7,8]	[9,10]	[11]	[12]	[13,14]	[15]	[16,17]
	Effectors		AMP, G6P	AMP, G6P	AMP, G6P	AMP	AMP, R5P	AMP, R5P	AMP, R5P	AMP	G6P	G6P, R5P, G3P	G6P
PEP	None	$S_{0.5}$ (mM)	0.41±0.01	1.0±0.1	1.0	1.2	0.24	0.96±0.09	6.6±1.0	0.24	N.A.	0.54	0.75±0.051
		h	1.82±0.05	2.04±0.4	3.7	2.0	1.5	2.24±0.37	2.8±0.4	1.6	N.A.	2.6	1.71±0.17
		k_{cat} (s^{-1})	182.9±1.2	63±4	N.A.	588	n.d.	187	97.6±14.8	n.d.	N.A.	238‡	39.1‡
		$k_{cat}/S_{0.5}$	446	63	N.A.	490	n.d.	194	14.7	n.d.	N.A.	440	52
	AMP	$S_{0.5}$ (mM)	0.25±0.01	N.A.	0.5	0.4	N.A.	0.28±0.05	0.6±0.0	N.A.	N.A.	N.A.	N.A.
		h	1.21±0.04	N.A.	1.5	1.1	N.A.	1.0	1.6±0.1	N.A.	N.A.	N.A.	N.A.
		k_{cat} (s^{-1})	194.8±2.1	N.A.	N.A.	690	N.A.	187	181±3	N.A.	N.A.	N.A.	N.A.
		$k_{cat}/S_{0.5}$	780	N.A.	N.A.	1725	N.A.	667	301	N.A.	N.A.	N.A.	N.A.
	G6P	$S_{0.5}$ (mM)	0.18±0.01	N.A.	0.47	N.A.	0.06 (R5P)	0.13±0.02 (R5P)	N.A.	N.A.	1.1	0.074	0.12±0.008
		h	1.24±0.07	N.A.	2.33	N.A.	N.A.	N.A. (R5P)	N.A.	N.A.	1.0	1.0	1.45±0.11
		k_{cat} (s^{-1})	172.7±2.8	N.A.	N.A.	N.A.	N.A.	187	N.A.	N.A.	113‡	261‡	40.9‡
		$k_{cat}/S_{0.5}$	960	N.A.	N.A.	N.A.	N.A.	1438 (R5P)	N.A.	N.A.	102	3527	340
	AMP/G6P	$S_{0.5}$ (mM)	0.18±0.01	n.d.	n.d.	N.A.	N.A.	N.A.	N.A.	N.A.	N.A.	N.A.	N.A.
		h	1.23±0.03	n.d.	n.d.	N.A.	N.A.	N.A.	N.A.	N.A.	N.A.	N.A.	N.A.
		k_{cat} (s^{-1})	188.8±1.4	n.d.	n.d.	N.A.	N.A.	N.A.	N.A.	N.A.	N.A.	N.A.	N.A.
		$k_{cat}/S_{0.5}$	1049	n.d.	n.d.	N.A.	N.A.	N.A.	N.A.	N.A.	N.A.	N.A.	N.A.
	ATP	$S_{0.5}$ (mM)	2.20±0.08	n.d.	N.A.	N.A.	N.A.	N.A.	N.A.	N.A.	N.A.	N.A.	N.A.
		h	2.63±0.21	n.d.	N.A.	N.A.	N.A.	N.A.	N.A.	N.A.	N.A.	N.A.	N.A.
		k_{cat} (s^{-1})	100.7±2.7	n.d.	N.A.	N.A.	N.A.	N.A.	N.A.	N.A.	N.A.	N.A.	N.A.
		$k_{cat}/S_{0.5}$	46	n.d.	N.A.	N.A.	N.A.	N.A.	N.A.	N.A.	N.A.	N.A.	N.A.
	ATP/AMP/G6P	$S_{0.5}$ (mM)	0.37±0.01	n.d.	N.A.	N.A.	N.A.	N.A.	N.A.	N.A.	N.A.	N.A.	N.A.
		h	1.15±0.03	n.d.	N.A.	N.A.	N.A.	N.A.	N.A.	N.A.	N.A.	N.A.	N.A.
		k_{cat} (s^{-1})	135.8±1.3	n.d.	N.A.	N.A.	N.A.	N.A.	N.A.	N.A.	N.A.	N.A.	N.A.
		$k_{cat}/S_{0.5}$	367	n.d.	N.A.	N.A.	N.A.	N.A.	N.A.	N.A.	N.A.	N.A.	N.A.
ADP	None	K_m (mM)	0.47±0.02	0.81±0.1	0.66	0.07	0.08	n.d.	0.4±0.1	0.16	0.39	0.095	0.18±0.11
AMP	None	$Ka_{0.5}$ (μM)	63.5±2.9	N.A.	500	n.d.	200	13	14†	n.d.	N.A.	N.A.	N.A.
	25 μM G6P	$Ka_{0.5}$ (μM)	35.7±0.9	n.d.	n.d.	N.A.	N.A.	N.A.	N.A.	n.d.	N.A.	N.A.	N.A.
	50 μM G6P	$Ka_{0.5}$ (μM)	20.3±1.4	n.d.	n.d.	N.A.	N.A.	N.A.	N.A.	n.d.	N.A.	N.A.	N.A.
G6P	None	$Ka_{0.5}$ (μM)	146.7±5.8	N.A.	240	N.A.	1 (R5P)	7.5 (R5P)	N.A.	N.A.	n.d.	3.7	n.d.
	12.5 μM AMP	$Ka_{0.5}$ (μM)	55.4±3.1	n.d.	n.d.	N.A.	N.A.	N.A.	N.A.	N.A.	N.A.	N.A.	N.A.
	25.0 μM AMP	$Ka_{0.5}$ (μM)	42.2±3.4	n.d.	n.d.	N.A.	N.A.	N.A.	N.A.	N.A.	N.A.	N.A.	N.A.

Mtb, Mycobacterium tuberculosis; Ms, Mycobacterium smegmatis; Cg, Corynebacterium glutamicum; Ec, Escherichia coli; Gs, Geobacillus stearothermophilus; Sa, Staphylococcus aureus; St, Salmonella typhimurium; Sm, Streptococcus mutans; Ss, Synechococcus sp.; Tg, Toxoplasma gondii

AMP, adenosine monophosphate; G6P, glucose 6-phosphate; R5P, ribose 5-phoshpate; G3P, glycerol 3-phosphate

$k_{cat}/S_{0.5}$ values in $s^{-1}\cdot mM^{-1}$; h, Hill coefficient; n.d., not done

N.A., not applicable (Either the molecule is not the modulator of the corresponding PYK, or the value is not indicated in the reference)

* The concentrations of modulators in the kinetic study of PEP are: AMP- 1 mM, G6P- 1 mM, ATP- 2mM

† Value is EC_{50} number

‡ k_{cat} is calculated assuming a subunit with mass of 54,366 g mol^{-1} (*Streptococcus mutans*), 63,212g mol^{-1} (*Synechococcus sp.*), and 55,863g mol^{-1} (*Toxoplasma gondii*)

Supplementary Table 2. Pairwise protein sequence comparisons of PYKs showing amino-acid identities

	*Mtb*PYK	*Ms*PYK	*Cg*PYK	*Ec*PYK-II	*Gs*PYK	*Sa*PYK	*St*PYK-II	*Sm*PYK	*Ss*PYK	*Tg*PYK-I
*Mtb*PYK		86.4	71.8	38.2	35.7	32.5	37.9	34.6	39.9	34.7
*Ms*PYK	86.4		72.2	38.4	35.7	33.6	38.4	34.4	40.6	36.2
*Cg*PYK	71.8	72.2		39	36.2	34.2	39	36	37.9	35.6
*Ec*PYK-II	38.2	38.4	39		39.8	38.7	98.5	30.3	39.6	34.3
*Gs*PYK	35.7	35.7	36.2	39.8		60.2	39.5	43.5	49.1	45.9
*Sa*PYK	32.5	33.6	34.2	38.7	60.2		38.3	41.8	44.4	43.2
*St*PYK-II	37.9	38.4	39	98.5	39.5	38.3		30.3	39.6	34.3
*Sm*PYK	34.6	34.4	36	30.3	43.5	41.8	30.3		36.5	37.4
*Ss*PYK	39.9	40.6	37.9	39.6	49.1	44.4	39.6	36.5		38.6
*Tg*PYK-I	34.7	36.2	35.6	34.3	45.9	43.2	34.3	37.4	38.6	

The pairwise sequence analysis was obtained from the program ClustalW2[1,2].
Values are overall percent sequence identities. The percentage values that over 60% are highlighted in yellow.
Mtb, Mycobacterium tuberculosis (UniProtKB: P9WKE5); *Ms, Mycobacterium smegmatis* (UniProtKB: A0QXA3); *Cg, Corynebacterium glutamicum* (UniProtKB: Q46078); *Ec, Escherichia coli* (UniProtKB: P21599); *Gs, Geobacillus stearothermophilus* (UniProtKB: Q02499); *Sa, Staphylococcus aureus* (UniProtKB: Q6GG09); *St, Salmonella typhimurium* (UniProtKB: Q8ZNW0); *Sm, Streptococcus mutans* (UniProtKB: Q8DTX7); *Ss, Synechococcus sp.* (UniProtKB: A0A0H3K2W0); *Tg, Toxoplasma gondii* (UniProtKB: Q969A2)

Supplementary Table 3. The AC-core motions during T- and R-state transition of the *Mtb*PYK tetramer

Reference structure (T-state)	Target structure (R-state)	AC-core rotation angle*	Average RMS† (Å)
T-state *Mtb*PYK	*Mtb*PYK-OX	8.2°±0.8°	2.8 (0.7)
T-state *Mtb*PYK	*Mtb*PYK-OX/ATP/AMP	8.7°±0.8°	3.1 (1.0)
T-state *Mtb*PYK	*Mtb*PYK-OX/G6P	9.1°±0.8°	3.3 (1.0)
T-state *Mtb*PYK	*Mtb*PYK-OX/AMP/G6P	9.0°±0.8°	3.2 (1.1)
T-state *Mtb*PYK	*Mtb*PYK-OX/AMP/G6P (soak)‡	9.0°±0.8°	3.3 (1.1)

* Rotation angle = value ± SEM
† Average Cα RMS difference from a tetramer superposition excluding the B domain; values of Cα RMS difference from a monomer superposition excluding the B domain shown in parentheses.
‡ Structure was determined from a *Mtb*PYK-OX crystal soaked with AMP and G6P.

Supplementary Table 4. Interface areas of *Mtb*PYK tetramers

*Mtb*PYK tetramer	A-A interface (Å^2)	C-C interface (Å^2)
T-state *Mtb*PYK	2181.1	2237.9
*Mtb*PYK-OX	3150.0	1715.8
*Mtb*PYK-OX/ATP/AMP	3185.3	1878.6
*Mtb*PYK-OX/G6P	3334.3	2004.6
*Mtb*PYK-OX/AMP/G6P	3298.2	1989.5
*Mtb*PYK-OX/AMP/G6P (soak)[†]	3343	1961.4

The interface areas are calculated via the online service PDBePISA[18].
† Structure was determined from a *Mtb*PYK-OX crystal soaked with AMP and G6P.

Supplementary Table 5. B-domain movements in different ligated states of *Mtb*PYK

*Mtb*PYK monomer	T-state *Mtb*PYK	
	Subunit A/B†	Subunit C/D
*Mtb*PYK-OX	18.6°	18.6°
*Mtb*PYK-OX/ATP/AMP	17.7°	20.2°
*Mtb*PYK-OX/G6P	17.6°	21.2°
*Mtb*PYK-OX/AMP/G6P	18.2°	21.2°
*Mtb*PYK-OX/AMP/G6P (soak)†	17.7°	21.7°

The closures of the B-domain from the T-state structure to the R-state structures were analysed using the Protein Domain Motion Analysis server DynDom[19,20]. The bending residues are Ile73-Gly76 and Ser158-Pro160.

† Subunits A/B/C/D are crystallographically independent chains and form a complete tetramer in an asymmetric unit. In each tetramer structure of *Mtb*PYK-OX/ATP/AMP, *Mtb*PYK-OX/G6P, *Mtb*PYK-OX/AMP/G6P and *Mtb*PYK-OX/AMP/G6P (soak), subunits A and B adopt a different B-domain position compared with subunits C and D. In the T-state structure and the MtbPYK-OX complex structure, all four chains of a tetramer have identical B-domain positions.

Supplementary Table 6. Codon-optimised gene of *Mtb*PYK for protein expression in *E.coli*

Gene	DNA sequence (5’-3’)
*Mtb*PYK	ATGACCCGTCGTGGCAAGATCGTTTGCACCCTGGGTCCGGCGACCCAGCGTGACGATCTGGTTCG TGCGCTGGTTGAGGCGGGTATGGACGTTGCGCGTATGAACTTCAGCCACGGCGATTACGACGATC ACAAGGTGGCGTATGAGCGTGTTCGTGTTGCGAGCGATGCGACCGGTCGTGCGGTGGGCGTTCTG GCGGATCTGCAGGGTCCGAAAATCCGTCTGGGTCGTTTTGCGAGCGGTGCGACCCACTGGGCGGA GGGTGAAACCGTTCGTATTACCGTGGGTGCGTGCGAAGGCAGCCATGACCGTGTTAGCACCACCT ATAAACGTCTGGCGCAAGATGCGGTGGCGGGTGATCGTGTGCTGGTTGACGATGGCAAAGTTGCG CTGGTTGTGGACGCGGTGGAGGGTGACGATGTGGTTTGCACCGTGGTTGAAGGTGGCCCGGTTAG CGATAACAAGGGTATCAGCCTGCCGGGTATGAACGTGACCGCGCCGGCGCTGAGCGAGAAAGAC ATTGAGGACCTGACCTTCGCGCTGAACCTGGGTGTGGACATGGTTGCGCTGAGCTTTGTGCGTAGC CCGGCGGACGTGGAGCTGGTTCACGAAGTGATGGATCGTATCGGCCGTCGTGTGCCGGTTATTGCG AAGCTGGAGAAACCGGAAGCGATCGATAACCTGGAGGCGATTGTTCTGGCGTTCGACGCGGTGAT GGTTGCGCGTGGTGATCTGGGCGTGGAACTGCCGCTGGAGGAAGTTCCGCTGGTGCAGAAGCGTGC GATCCAAATGGCGCGTGAGAACGCGAAACCGGTGATCGTTGCGACCCAAATGCTGGACAGCATGAT TGAGAACAGCCGTCCGACCCGTGCGGAAGCGAGCGATGTTGCGAACGCGGTGCTGGACGGTGCGGA TGCGCTGATGCTGAGCGGTGAAACCAGCGTTGGTAAATACCCGCTGGCGGCGGTGCGTACCATGAG CCGTATCATTTGCGCGGTTGAAGAAAACAGCACCGCGGCGCCGCCGCTGACCCACATTCCGCGTAC CAAGCGTGGTGTGATCAGCTATGCGGCGCGTGACATTGGCGAGCGTCTGGATGCGAAAGCGCTGGT TGCGTTTACCCAAAGCGGTGACACCGTGCGTCGTCTGGCGCGTCTGCATACCCCGCTGCCGCTGCTG GCGTTCACCGCGTGGCCGGAAGTGCGTAGCCAACTGGCGATGACCTGGGGCACCGAAACCTTTATC GTTCCGAAAATGCAGAGCACCGACGGCATGATTCGTCAAGTGGATAAGAGCCTGCTGGAACTGGCG CGTTATAAACGTGGTGATCTGGTGGTTATTGTTGCGGGTGCTCCGCCGGGCACCGTGGGCAGCACCA ACCTGATCCACGTTCACCGTATTGGCGAGGACGATGTGTAA

Supplementary References

1. Thompson, J. D., Higgins, D. G. & Gibson, T. J. CLUSTAL W: improving the sensitivity of progressive multiple sequence alignment through sequence weighting, position-specific gap penalties and weight matrix choice. *Nucl. Acids Res.* **22,** 4673–4680 (1994).
2. Larkin, M. A. *et al.* Clustal W and Clustal X version 2.0. *Bioinformatics* **23,** 2947–2948 (2007).
3. Kearse, M. *et al.* Geneious Basic: An integrated and extendable desktop software platform for the organization and analysis of sequence data. *Bioinformatics* **28,** 1647–1649 (2012).
4. Noy, T. *et al.* Central Role of Pyruvate Kinase in Carbon Co-Catabolism of Mycobacterium tuberculosis. *J. Biol. Chem.* **291,** 7060–7069 (2016).
5. Kapoor, R. & Venkitasubramanian, T. A. Glucose 6-phosphate activation of pyruvate kinase from Mycobacterium smegmatis. *Biochemical Journal* **193,** 435–440 (1981).
6. Jetten, M. S., Gubler, M. E., Lee, S. H. & Sinskey, A. J. Structural and functional analysis of pyruvate kinase from Corynebacterium glutamicum. *Appl. Environ. Microbiol.* **60,** 2501–2507 (1994).
7. Waygood, E. B., Rayman, M. K. & Sanwal, B. D. The Control of Pyruvate Kinases of Escherichia coli. II. Effectors and Regulatory Properties of the Enzyme Activated by Ribose 5-Phosphate. *Canadian Journal of Biochemistry* **53,** 444–454 (1975).
8. Waygood, E. B. & Sanwal, B. D. The control of pyruvate kinases of Escherichia coli. I. Physicochemical and regulatory properties of the enzyme activated by fructose 1,6-diphosphate. *J. Biol. Chem.* **249,** 265–274 (1974).
9. Suzuki, K., Ito, S., Shimizu-Ibuka, A. & Sakai, H. Crystal structure of pyruvate kinase from Geobacillus stearothermophilus. *J. Biochem.* **144,** 305–312 (2008).
10. Sakai, H. Possible Structure and Function of the Extra C-Terminal Sequence of Pyruvate Kinase from Bacillus stearothermophilus. *J. Biochem.* **136,** 471–476 (2004).
11. Zoraghi, R. *et al.* Functional Analysis, Overexpression, and Kinetic Characterization of Pyruvate Kinase from Methicillin-Resistant Staphylococcus aureus. *Biochemistry* **49,** 7733–7747 (2010).
12. Garcia-Olalla, C. & Garrido-Pertierra, A. Purification and kinetic properties of pyruvate kinase isoenzymes of Salmonella typhimurium. *Biochemical Journal* **241,** 573–581 (1987).
13. Yamada, T. & Carlsson, J. Glucose-6-phosphate-dependent pyruvate kinase in Streptococcus mutans. *J. Bacteriol.* **124,** 562–563 (1975).
14. Abbe, K. & Yamada, T. Purification and properties of pyruvate kinase from Streptococcus mutans. *J. Bacteriol.* **149,** 299–305 (1982).
15. Knowles, V. L., Smith, C. S., Smith, C. R. & Plaxton, W. C. Structural and regulatory properties of pyruvate kinase from the Cyanobacterium synechococcus PCC 6301. *J. Biol. Chem.* **276,** 20966–20972 (2001).
16. Maeda, T. *et al.* Expression and characterization of recombinant pyruvate kinase from Toxoplasma gondii tachyzoites. *Parasitol Res* **89,** 259–265
17. Bakszt, R. *et al.* The crystal structure of Toxoplasma gondii pyruvate kinase 1. *PLoS ONE* **5,** e12736 (2010).
18. Krissinel, E. & Henrick, K. Inference of macromolecular assemblies from crystalline state. *J. Mol. Biol.* (2007).
19. Hayward, S. & Berendsen, H. J. C. Systematic analysis of domain motions in proteins from conformational change: New results on citrate synthase and T4 lysozyme. *Proteins* **30,** 144–154 (1998).
20. Hayward, S., Kitao, A. & Berendsen, H. J. C. Model-free methods of analyzing domain motions in proteins from simulation: A comparison of normal mode analysis and molecular dynamics simulation of lysozyme. *Proteins* **27,** 425–437 (1997).
