# Supplementary material for: Allosteric pyruvate kinase-based “logic gate” synergistically senses energy and sugar levels in Mycobacterium tuberculosis
Source: Nat Commun. 2017 Dec 7;8:1986. doi: 10.1038/s41467-017-02086-y (PMC5719368; doi:10.1038/s41467-017-02086-y)
Supplement: Supplementary file 2 — Description of Additional Supplementary Files [file 41467_2017_2086_MOESM2_ESM.pdf]

Description of Supplementary Files

File Name: Supplementary Movie 1
Description: **Rigid-body rotations in T- to R-state transition.** Individual chains are highlighted (brown, cyan, blue and yellow), while effectors (AMP and G6P) and active-site ligands (Mg2+ and oxalate) are shown by spheres.

File Name: Supplementary Movie 2
Description: **Structural interplay across the C-C interface in response to the T to R transition.** A close-up view of the C-C interface highlights conformational changes and side chain movements that occur as the protomers rotate from T- to R- (AMP/G6P-bound) state. Effectors (AMP and G6P) are shown by spheres and hydrogen bonds are shown as dashed lines. Key residues involved in interface interactions are shown as sticks. Despite the distinct evolutionary divergence between *Mtb*PYK and human M2PYK, the role of the effector loop is similar whereby the effector loop forms a salt bridge and hydrogen bond across the C-C interface in the phenylalanine-bound T-state human M2PYK but swivels out from the C-C interface to interact with F16BP at the allosteric site in the F16BP-bound R-state enzyme.

File Name: Supplementary Movie 3
Description: **Allosteric-site plasticity of *Mtb*PYK.** An enlargement of the allosteric sites highlights structural rearrangements that occur as effectors AMP and G6P allosterically activate the protein. Effectors (AMP and G6P) are shown by spheres and key moving residues are shown as sticks.
